# The role of manganese-based MRI contrast agents for cancer theranostics: Where do we stand in 2025?

**DOI:** 10.7150/thno.108705

**Published:** 2025-03-15

**Authors:** Lingyan Zhang, Shubham Roy, Bing Guo

**Affiliations:** 1 Lab of Molecular Imaging and Medical Intelligence, Department of Radiology, Longgang Central Hospital of Shenzhen, Shenzhen, 518116, China.; 2 Shenzhen Clinical Medical College, Guangzhou University of Chinese Medicine, Shenzhen, Shenzhen, 518116, China.; 3 Longgang Clinical Institute of Shantou University Medical College Shenzhen, 518116, China.; 4 School of Science, Shenzhen Key Laboratory of Advanced Functional Carbon Materials Research and Comprehensive Application, Harbin Institute of Technology, Shenzhen, 518055, China.

**Keywords:** Manganese, Cancer, Magnetic resonance imaging, Nanotheranostics, Combinatory therapy

## Abstract

Magnetic resonance imaging (MRI) guidance in the realm of anticancer therapy is crucial to visualize the spread of tumors in deep tissues, accumulate the therapeutics, and trigger them for precise therapy. Recent studies bridge this gap by integrating MRI contrast agents (CAs) with different therapeutic regimes for a better outcome. In this context, manganese-based materials hold great potential owing to their *T_1_/T2* dual-modal MR-relaxation, less toxicity, and other therapeutic capabilities such as chemodynamic therapy, immunotherapy, etc., which have gained increasing interest among researchers and medical professionals. This work offers a timely update on the last three years for Mn-based MRI-guided theranostic applications, including chemodynamic therapy, chemotherapy, photo therapies, sonodynamic therapy, and radiotherapy against cancer. Further, several combinatory therapies and surgical intersections have also been summarized in the light of MRI guidance. The design rationale of these Mn-based agents has been discussed to understand the existing challenges and plausible outcomes shortly. Herein, we deep dive into the stimulus-based probes including pH, temperature, etc. to show the unexplored potential of Mn-complexes in this domain. This state-of-the-art review will guide innovations in Mn-based CAs to expedite safer anticancer theranostic modules for clinical translation.

## 1. Introduction

Cancer has caused severe harm to human health by spreading exponentially worldwide irrespective of the age of the patient. Recent studies highlight several therapeutic modalities that can cure cancer to a certain level and extend the life expectancy of a patient. However, detecting cancer is crucial to inhibit its spread and start the therapy in time 1. It is observed that probing the cancer in time could expedite the therapeutic capability and restrict metastasis 1. Thus, a combination of precise therapy with diagnosis is needed for an advanced therapeutic regimen. Currently, theranostic nanoplatforms (especially imaging-guided delivery systems) combining therapeutic and diagnostic modalities have shown promise for cancer treatment 2-3. The real-time visualization of the therapeutics, accumulation of them in the diseased tissues, and the outcomes of the therapy make these systems advanced compared to conventional chemotherapy and immunotherapy alone 4. Recently, fluorescence imaging (FI), magnetic resonance imaging (MRI), computed tomography (CT), X-ray imaging, etc. have become important modalities owing to their high signal-to-noise ratio (SNR), higher tissue penetration, and low autofluorescence 5. Although FI in the NIR-II domain can penetrate up to several millimeters and image soft tissues and veins quite clearly, they have limited accuracy when subjected to bone imaging. On the other hand, X-rays and CT can precisely detect bone lesions, but the use of ionizing radiations and toxic contrast agents (CAs) limits their biocompatibility 6. On the other hand, MRI can detect infections both in soft tissues and bone with high accuracy and utilizes the magnetic response of the CAs without damaging any cells or tissues. More importantly, it can penetrate through skin and tissues to the highest depth 7. Thus, MRI has become a favorable choice to image cancer margins even in deep tissues 8-9.

Paramagnetic materials as contrast agents are popular owing to their stronger magnetic properties. The number of unpaired electrons in the Gd^3+^ ion is 7, whereas, for Mn^2+^, the number of unpaired electrons is 5 11. As the number of unpaired electrons determines the magnetic moment, Gd^3+^ possesses a higher magnetic moment with better MR imaging capability. MRI contrast agents have higher relaxation rates (*T1* and *T2*) of nearby water protons. Mn ions (Mn^2+^) are highly efficient at *T1*-weighted contrast enhancement, leading to bright images. Free Gd^3+^ ions are highly toxic because they interfere with calcium-dependent biological processes. To mitigate this, Gd^3+^ is used in chelated forms, such as Gd-DTPA 10. The gadolinium deposition in tissues, especially in the brain, can cause massive toxicity. Gd is a heavy metal, and in its free ionic form, it is highly toxic to human tissues. When the kidneys process Gd-based contrast agents (GBCAs), they are exposed to potential toxicity, especially in patients with pre-existing kidney impairment 11. Excessive or unregulated doses of gadolinium (Gd) can lead to severe kidney damage, including conditions like nephrogenic systemic fibrosis (NSF) in some cases. This highlights the critical need for a safer and more biocompatible alternative, such as contrast agents (CA), to minimize potential risks while ensuring effective diagnostic imaging 12. Mn, on the other hand, functions as both a *T1* and a *T2* contrast agent in magnetic resonance imaging (MRI), demonstrating dual-mode functionality 13. The magnetic characteristics of contrast agents, such as manganese (Mn), affect the relaxation times (*T1*) (longitudinal) and (*T2*) (transverse) of the surrounding water protons. A powerful local magnetic field produced by Mn's unpaired electrons interacts with the nuclear spins of neighboring water protons. Because Mn effectively promotes energy transfer from excited protons to their surroundings, *T1* relaxation, which is linked to increased signal intensity in *T1*-weighted MRI is shortened. Conversely, field inhomogeneities of Mn also impact *T2* relaxation, which is associated with the loss of phase coherence among proton spins and results in signal decay in *T2*-weighted MRI 14. These effects, which allow for customized imaging contrast for certain diagnostic requirements, are dependent on Mn content, particle size, and surface chemistry. To improve image quality in magnetic resonance imaging (MRI), gadolinium-based contrast agents (GBCAs) minimize the relaxation durations *T1* of surrounding water protons. The seven unpaired electrons in gadolinium (Gd³⁺) produce a powerful magnetic moment that interacts with neighboring protons. In *T1*-weighted images, this interaction increases the signal intensity and speeds up *T1* relaxation, highlighting pathological and anatomical details. Usually chelated with ligands to lower toxicity, Gd³⁺ ensures safe excretion while retaining its effectiveness. When it comes to finding anomalies in the central nervous system, tumors, and blood arteries, GBCAs are especially useful 15. Yet, worries about long-term tissue preservation have spurred research into less harmful substitutes, like manganese-based contrast agents, which have comparable advantages but less toxicity.

Mn can induce Fenton-like reactions and initiate the cGAS-STING pathway to promote chemodynamic therapy (CDT) and immunotherapy 16. Moreover, Mn can be coordinated with organic dyes like porphyrins to yield MRI-functionalized dyes for various applications such as MRI-guided PTT, and sonodynamic therapy. Additionally, it can be doped/mixed in nanoplatforms via covalent and/or non-covalent linkages yielding multiple functional theranostic nanomedicines with MRI guidance. These features enable Mn to serve both imaging and therapeutic purposes in cancer. **Table 1** compares the (*r_1_*) relaxivity of typical gadolinium-based agents with manganese-based contrast agents, highlighting that Mn-based agents exhibit a higher relaxivity than Gd agents.

Under specific circumstances, Mn-based treatments can also produce reactive oxygen species (ROS), which can help destroy cancer cells. 30. Furthermore, Mn can enhance immune responses against tumors by activating the cGAS-STING immunological system, which increases immunological responses by inducing the production of pro-inflammatory cytokines and type I interferons 31. Transforming hydrogen peroxide into oxygen, Mn-based treatments can improve oxygen availability and boost therapeutic efficacy while reducing tumor hypoxia 32. Manganese (Mn) improves cancer treatment through a variety of interactions with the tumor microenvironment (TME) 33. By causing oxidative stress and producing reactive oxygen species (ROS), which harm cancer cells and encourage cell death, Mn^2+^ ions can alter the TME. Mn interacts with the TME to facilitate medication transport and treatment responses, in part because of its capacity to modify pH, control the tumor extracellular matrix, and influence redox processes 34. Being a common nutrient, Mn is a safer substitute compared to traditional agents due to its biodegradability and low toxicity. By reducing off-target effects and increasing therapeutic accuracy, Mn-based compounds can be designed for tailored drug delivery. Besides, Mn promotes drug distribution and penetration by modifying the extracellular matrix and controlling pH levels. The potential of Mn-based contrast agents to enhance imaging and therapeutic precision in cancer treatment is highlighted by these interactions. Furthermore, Mn-based medicines can be used with immunotherapy, photothermal therapy, chemotherapy, and other treatments to provide a multimodal strategy for more successful cancer treatment 35.

Apart from the therapeutic or diagnostic efficacy of Mn, in its ionic form Mn can detect cancer in several ways. Notably, cancer cells can uptake Mn ions due to their strong affinity towards Mn. Manganese chloride (Mn chloride), specifically the Mn²⁺, enters cells via calcium channels. However, its application in cancer research has been relatively limited. In breast cancer, studies using MCF7 and MDA-MB-231 tumor models demonstrated that areas of significant Mn enhancement on MRI correlated with strong CaSR expression. However, these studies did not compare enhancement levels between the two cell lines, nor did they quantify Mn uptake 36. Mn interacts with the tumor microenvironment (TME) significantly to improve cancer treatment 37. MnO_2_ in the nanoparticle form can be catalyzed through the tumor microenvironment by the local H_2_O_2_ and internalized by the cancer cells in many works 38. These works greatly support the importance of Mn-based systems in cancer theranostics.

**Figure 1:**
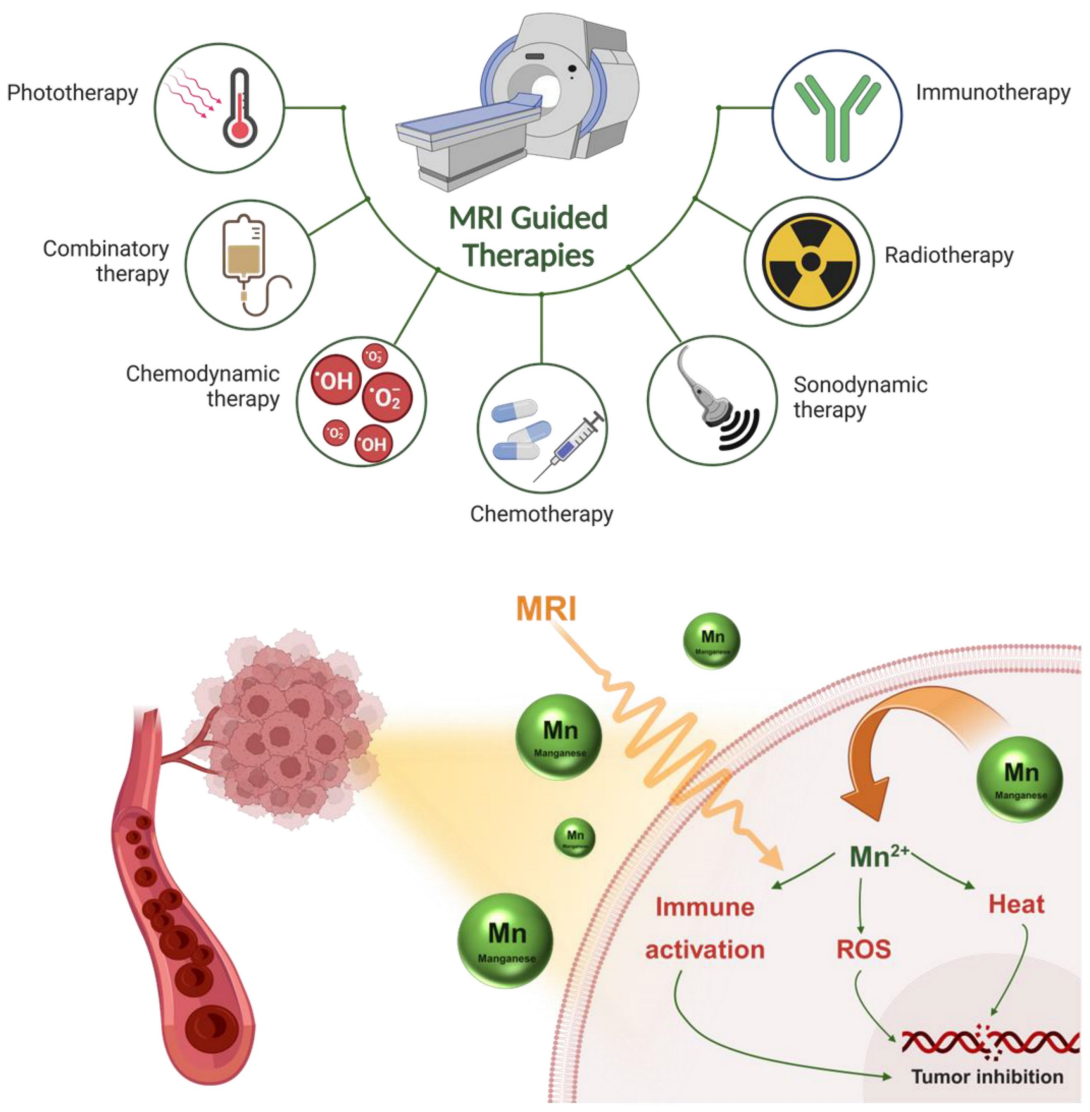
Manganese-based MRI-guided therapies like immunotherapy, radiotherapy, sonodynamic therapy, chemotherapy, phototherapy, etc. for cancer theranostics. This figure also illustrates different pathways involving Mn to promote ROS, heat, and immunity-based activation processes in a biological system for anticancer therapy.

Very recently the emergence of Mn-based small molecules, polymer dots, quantum dots, and biomimetic NPs made these systems very efficient and provided maximum theranostic efficacy **(Figure 1)**. Mn can be tumor-microenvironment (TME) responsive and stimulate different pathways to achieve such functionalities. Various Mn-based systems and their applications have been reviewed to modulate the TME. These methods include producing reactive oxygen species, increasing pH, oxygen generation, glutathione/hydrogen peroxide depletion, glucose exhaustion, and activating innate or adaptive immunity 39. Increased imaging signals always accompany all of these mechanisms for response monitoring. Due to these significant benefits, Mn-based systems have a wide range of uses in TME-responsive cancer therapies, such as chemotherapy, gene therapy, gas therapy, immunotherapy, photodynamic therapy (PDT), sonodynamic therapy (SDT), radiotherapy, starvation therapy, chemodynamic therapy (CDT), ferroptosis-mediated therapy, and immunotherapy 40. There are several reviews highlighting Mn-based STING immunotherapy, Mn as MRI agents, designing rationale of Mn-based contrast agents, etc till the end of the last decade 41. These reviews also described cancer treatments that include heat production in TME, such as photothermal therapy (PTT), microwave dynamic therapy (MDT), magnetic hyperthermia, and microwave thermal therapy (MTT), paving a path to understanding innovative therapeutic approaches 32. Likewise, Huang *et al.* showed the physicochemical properties and synthesis techniques of biomaterials derived from Mn, which are thoroughly examined in his review article, with a focus on their application in tumor diagnostics, encompassing magnetic resonance imaging, photothermal and photoacoustic imaging, ultrasound imaging, multimodal imaging, and detection. The benefits of using Mn-based materials for tumor treatment applications are also covered. These applications include tumor immunotherapy, drug delivery, tumor microenvironment control, synergistic photothermal, photodynamic, and chemodynamic therapies, and imaging-guided therapy 42. However, scientists focus on increasing the efficiency of such contrast agents by making them 'all-in-one' theranostic systems for more effective and personalized cancer treatment. Notably, very recent research on Mn-based STING immunotherapy, Fenton reaction-based chemodynamic therapy, lipid peroxidation mediated anticancer therapy, and multimodal imaging-guided anticancer therapies have not been included in such review, creating a scope of further summarizing these new domains and analyzing their future prospects.

Herein, we offer a timely update on the recent progress, especially from 2022 to the present time, on how to design Mn-based theranostic platforms for optimal efficacy. This work also highlights the novelty of manganese-based agents in MRI-guided theranostics by emphasizing their *T1/T2* dual-modal MR imaging capabilities, superior biocompatibility, and multifunctional therapeutic potential. Unlike conventional MRI contrast agents, Mn-based systems provide enhanced tumor visualization and precise therapeutic triggering, bridging imaging with therapies like chemodynamic therapy, chemotherapy, and phototherapies. The integration of stimulus-responsive probes sensitive to pH, temperature, and other microenvironmental changes further distinguishes this approach, offering dynamic control over therapeutic delivery. Importantly, the combinatory therapeutic platforms with Mn-based MRI imaging guidance are also pointed out. Afterward, some key nanotoxicity challenges, potential obstacles, and prospects are also discussed. In the end, Mn-based nanomaterial-derived cancer nanomedicine is expected to progress in terms of development and prospects. By summarizing the latest advancements and addressing challenges in Mn-based theranostics, this review sets a foundation for innovative designs, ensuring safer and more effective cancer treatments for clinical translation.

## 2. Theranostic agents and their implications on biomedical applications

The concept known as "theranostic," which represents the combination of imaging and therapy, is referred to by several groups 43-46. It is described as the endeavor to combine investigative and therapeutic capabilities into a single agent to create personalized, targeted therapies for a range of diseases 47. John Funkhouser initially introduced this term, and it has more recently been found to be applicable in the context of image-guided therapy and therapeutic agents that also possess imaging capabilities. In the past, the conventional approach involved the diagnosis and subsequent therapy to address the specific disease. Consequently, medical research primarily concentrated on characterizing the ailment and creating standardized treatments or medications. However, some diseases like cancer, which exhibit diverse expressions, demand tailored and personalized treatment approaches 48. Theranostic strategies herein play a crucial role in developing advanced medical efficacy.

The rapidly advancing field of nanotheranostics is distinguished by its focus on drug delivery, drug release, and the assessment of therapeutic effectiveness, all of which are simultaneously monitored through a single nanoscale carrier 49. Nanoparticles within the range of 1 to 100 nm are believed to display discriminating interactions with biomolecules, including receptors, antibodies, and enzymes located either inside or outside of cells 50.

Nanotechnology not only elevates the performance of the theranostic systems but also introduces imaging guidance along with high therapeutic efficiencies. MRI-imaging guided therapy is one of such recent advancements 51. One of the significant contenders in creating theranostic nanoplatforms has been the transformation of MRI contrast agents (CAs). The precise delivery of contrast agents, like iron oxide NPs, Mn^2+^, and Gd^3+^ complexes, to the specific tumor site through custom-designed nanoplatforms such as block copolymers, liposomes, graphene oxide, and hydrogels, plays a vital role in conferring highly sensitive MRI contrast capabilities to the theranostic nanosystem 52.

Among these MRI theranostics systems, Mn-based MRI probes have become a prevalent choice owing to their inertness, higher biocompatibility, and lower cytotoxicity. In the following chapter such Mn-based MRI theranostic systems have been discussed.

## 3. Mn-based agents for MRI-guided theranostic applications

### 3.1 Mn-based contrast agents for MRI-guided chemotherapy

Chemotherapy is a medical therapy that employs medications to destroy or slow down the proliferation of cancer cells or other fast-growing cells in the body 53. Chemotherapy is a crucial therapeutic modality for cancer treatment 54. The side effects and overcoming drug resistance are critical factors. One of the primary factors contributing to this resistance is the overexpression of ATP-binding cassette (ABC) transporters on the surface of tumor cells, which could be minimized if other therapeutic modalities could be integrated into the existing chemotherapy 55. Herein, using Mn could be beneficial as it has multiple therapeutic and imaging abilities. Chemotherapeutic drugs such as doxorubicin, gemcitabine, and paclitaxel are commonly used in cancer treatment to reduce or eradicate tumors, frequently by focussing on rapidly proliferating cancer cells 56. Nevertheless, these medications are poorly soluble and have brief *in vivo* half-lives. Nanocarriers are employed to improve controlled release and focused distribution. Using manganese ions (Mn²⁺) to cause toxicity in cancer cells through redox processes, manganese-based chemotherapy is a novel strategy that aims to target tumors while minimizing harm to healthy organs efficiently 57.

The potential of manganese-based (Mn-based) contrast agents in MRI-guided chemotherapy is drawing more attention 58. By clearly visualising tumors, these medicines improve MRI imaging and allow for more accurate treatment planning and monitoring. Additionally, Mn-based compounds help improve drug administration and uptake, overcome obstacles including tumor hypoxia, and modify the tumor microenvironment 59. Mn-based drugs are promising options for maximizing chemotherapeutic efficacy and minimizing negative effects because of their dual role. MRI-guided chemotherapy can provide spatial information and show improved cancer therapeutic efficacy. The MRI-based chemotherapy follows the ''sense and release" approach, usually getting triggered under the tumor microenvironment 60. It has been established that Mn-MOF complexes possess a high affinity for tumor cells and can be considered a suitable choice for delivering chemotherapeutic drugs 61. For example, Song *et al.* loaded DOX into Mn nanoparticles to diagnose breast cancer cells in 4T1 mice and enhanced treatment via chemotherapy 62. In another study, Pan and colleagues created a multifunctional nanoplatform consisting of BSA-MnO_2_ (~10 nm) for tumor and renal imaging and chemotherapy. Indocyanine green and paclitaxel, which act as photothermal and chemotherapeutic agents, are loaded in the nanostructure 63. The T1 relaxivity was found to be 7.9 mM^-1^ s^-1^. Such work opens up new avenues for the development of new and integrated biomaterials for cancer therapy. Another biocompatible and biodegradable multifunctional integrated nano platform was reported by Zhou *et al.* 64. Researchers created Mn-doped calcium phosphate nanoparticles for targeted DOX delivery in MRI-guided cancer therapy. These nanoparticles demonstrated improved therapeutic effects against BxPC-3 cells and robust T1-MR relaxation, providing a viable method for imaging-guided cancer treatment 65.

**Figure 2:**
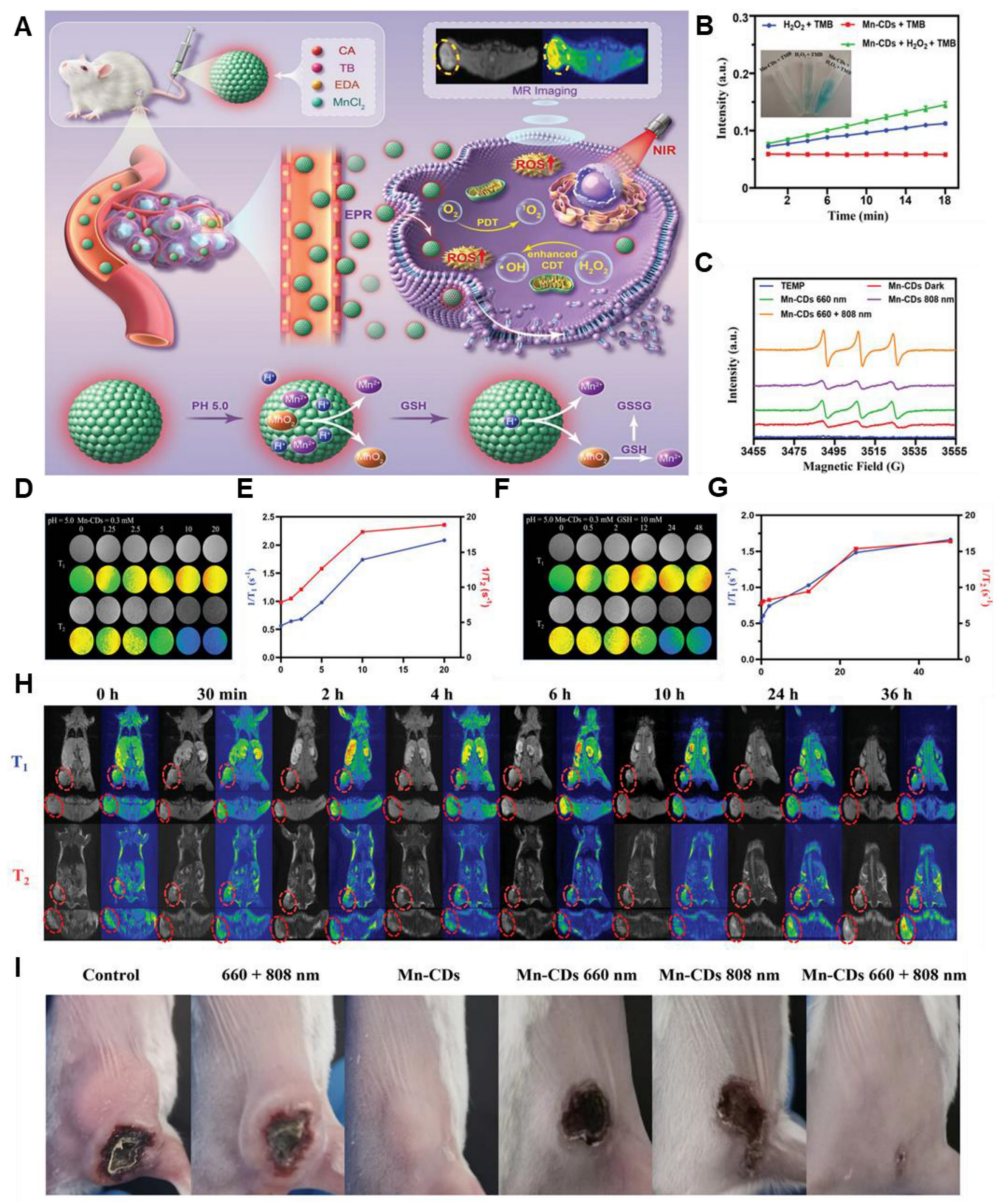
**(A)** Schematic illustration of Manganese-doped TB CDs for MRI-guided light-enhanced CDT and PDT. **(B)** Catalytic activity of Mn-CDs using TMB as the chromogenic substrate. **(C)** ESR spectra of Mn-CDs in aqueous solution under 660, 808, 660 + 808 nm laser irradiation. **(D-G)** *T1* and *T2* relaxation performance of the sample. **(H)** Cross-sectional and coronal *T1*- and *T2*-weighted pictures, as well as pseudocolor images, of the mice model before and after Mn-CD injection at various time points for 4*T1* tumors. **(I)** Pictures of the 4*T1 in vivo* tumor-bearing mice in each group after the therapy. Adapted with permission from 66, copyright 2023 Wiley-VCH.

Recently, by focussing on the tumor microenvironment (TME), researchers are improving MR relaxation rates and chemotherapeutic capabilities. For instance, carbon dot nanozymes based on manganese and toluidine blue (TB) demonstrated a 225-250% enhancement in dual *T1/T2* MR relaxation. Due to their increased permeability and retention effect, these enzymes also demonstrated peroxidase-like activity because of the Mn and were responsive to the TME, which encouraged aggregation at tumor locations **(Figure 2)** 66.

Nevertheless, by improving tumor imaging, facilitating targeted drug distribution, and increasing therapeutic efficacy, Mn-based contrast agents improve MRI-guided chemotherapy and present a viable strategy for integrated cancer treatment with few adverse effects.

### 3.2 Mn-based contrast agents for MRI-guided chemodynamic therapy

Chemodynamic therapy (CDT) is an emerging therapeutic approach in the field of cancer treatment that harnesses chemical reactions to selectively generate toxic species, such as reactive oxygen species (ROS), within tumor cells 67. Unlike traditional chemotherapy, which relies on delivering cytotoxic drugs, CDT exploits the unique biochemical and microenvironmental characteristics of cancer cells to induce cell death. The use of manganese-based (Mn-based) contrast agents for MRI-guided chemodynamic treatment (CDT) is becoming more popular 68. Their paramagnetic characteristics improve tumor localization and treatment planning by enabling accurate tumor imaging via MRI. Reactive oxygen species (ROS) are produced in the tumor microenvironment by Mn-based therapies, which promote oxidative stress and tumor cell death 69. Mn-based contrast agents are a promising technique for enhancing the efficacy of chemodynamic cancer therapies because of their dual functionality, which combines therapeutic activity with diagnostic imaging.

**Figure 3:**
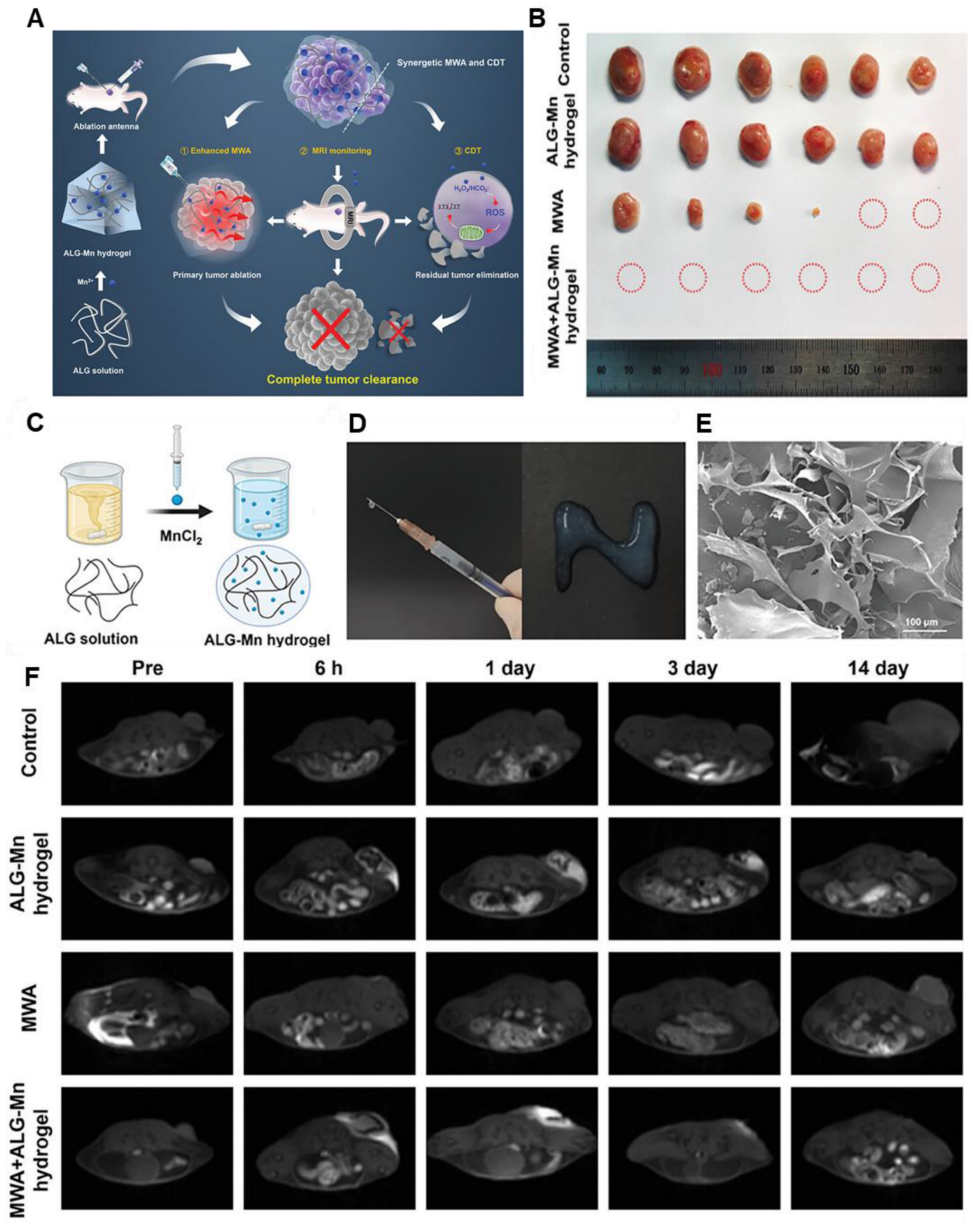
** (A)** The synthesis and mechanism of ALG-Mn hydrogel for synergistic MWA and CDT oncotherapy towards total tumor clearance are shown schematically. **(B)** Pictures of the tumor tissues taken from the mice on the 15th day following different treatments **(C)** Diagrammatic representation of the preparation of ALG-Mn hydrogel. **(D)** Assessment of the syringeability of ALG-Mn hydrogel, and **(E)** SEM images of the prepared hydrogel. **(F)** Different groups of *in vivo* MR images demonstrating the potential of the samples as an MRI agent. Adapted with permission from 72, copyright 2024 Wiley-VCH.

The outstanding efficacy of chemodynamic therapy has further been elevated to a new level when scientists attached the MRI imaging guidance. According to a recent study, Gao *et al.* synthesized a Pt-MnO_2_ complex that can perform synergistic chemo/chemo dynamic therapies for tumor cells. The tumor cells could specifically internalize L/D-MnO_2_@Pt NPs, and the efficient depletion of glutathione (GSH) through redox reaction was achieved, resulting in the release of Mn^2+^ and Pt. A strong chemodynamic effect was exhibited by the released Mn^2+^ through a Fenton-like reaction. The chemodynamic therapy (CDT) efficiency was further improved by the depletion of GSH. Such results in recent times provoked other groups to take up imaging-guided chemodynamic treatment of diseased organs and cells. For instance, Lin *et al.* designed a MnO_2_-based self-reinforcing CDT nano-agent (MS@MnO_2_) that can react with the GSH in the tumor site and produce GSSH, along with a reduction of MnO_2_ to Mn^2+^ 70. This Mn^2+^ catalyzes the •OH from H_2_O_2_ with high oxygen activity. This reactive •OH is toxic to the cancer cells as it hampers the DNA, proteins, and lipids of tumor cells, ultimately killing the cells. The Mn^2+^ ions also help in CDT tracking via MRI. He and his team developed metastable γ-MnS@BSA for MRI-guided CDT and simultaneous gas therapy 71. The Mn nanoparticle was synthesized via the wet chemical method. In the tumor site, Mn^2+^ and S^2-^ act as the donor of reactive species and H_2_S gas, respectively. At acidic pH, H_2_S gas increased to 12 μM, which is effective enough to kill the cancer cells. Although there are several limitations of chemodynamic therapy such as excessive ROS/RNS generation, uncontrolled reaction rate, etc. However, imaging guidance seems to have promising qualities to probe such inconsistencies making this modality a futuristic candidate in MRI-imaging-guided theranostic modules. Besides, several minimalistic approaches have been adopted by some groups to undergo tumor treatment **(Figure 3)** 72. Injectable hydrogels containing Mn have been used for this purpose. It is seen that the presence of Mn^2+^ not only promoted the entanglement of the hydrogel but also facilitated the Fenton reaction for a precise CDT. Moreover, it produced an enhanced *T1* MR relaxation for *in vivo* imaging of tumors. Thus, the use of Mn could aid not only in the Fenton reaction and MRI guidance but also in preparing the hydrogel matrix and integrating other therapies in a minimalistic way.

Therefore, through its ability to catalyze the Fenton-like reaction, which produces hydroxyl radicals, oxidative stress, and cancer cell damage, manganese (Mn) improves MRI-guided chemodynamic treatment (CDT). Mn^2+^ enhances CDT effects by decreasing tumor hypoxia, and its paramagnetic properties enhance *T1*-weighted MRI imaging for accurate tumor visualization and therapeutic tracking. Mn integrates therapeutic and diagnostic activities to effectively treat cancer by facilitating regulated drug release in the acidic tumor microenvironment.

### 3.3 Mn-based contrast agents for MRI-guided photothermal therapy

MRI-guided photothermal treatment (PTT) shows promise with manganese-based (Mn-based) contrast agents. For accurate tumor monitoring and localization, their paramagnetic qualities improve MRI imaging. When light is activated, Mn-based compounds can also produce heat, which effectively kills tumor cells and promotes photothermal effects. Furthermore, Mn ions improve therapy results by altering the tumor microenvironment. When imaging and therapy are combined, Mn-based compounds are a great option to improve the accuracy and effectiveness of photothermal cancer treatments. Phototheranostics or phototherapy typically involves the synergistic use of photosensitizers and light in cancer treatment. Photothermal therapy (PTT) is a form of hyperthermia treatment that entails subjecting bodily tissues to elevated temperatures above the normal range as part of cancer therapy 73. In most cases, NIR irradiation is used with photothermal agents that can potentially convert the light into heat. Photosensitizers should possess the property of moving to an excited state at a particular frequency and then releasing the energy as heat to kill the localized/targeted cells 74.

It is observed that photobleaching is a vital phenomenon that affects the overall optical properties of a system. For an efficient photostable system, photobleaching must be optimized. In this context, MacDonald *et al.* reported that Mn^3+^ ions incorporated building blocks of porphysome nanoparticles can efficiently resist photobleaching and thereby enhance the MRI signal. According to them, it can rival Gd-DTPA for MRI contrast generation. Apart from that, this sample can show stable photothermal efficacy under 680 nm laser (0.75 W) and effectively maintain over 40 °C for longer durations 75. These types of MRI-guided PTT agents could be effective in clinical research as they have efficient photostability along with bioavailability.

**Figure 4:**
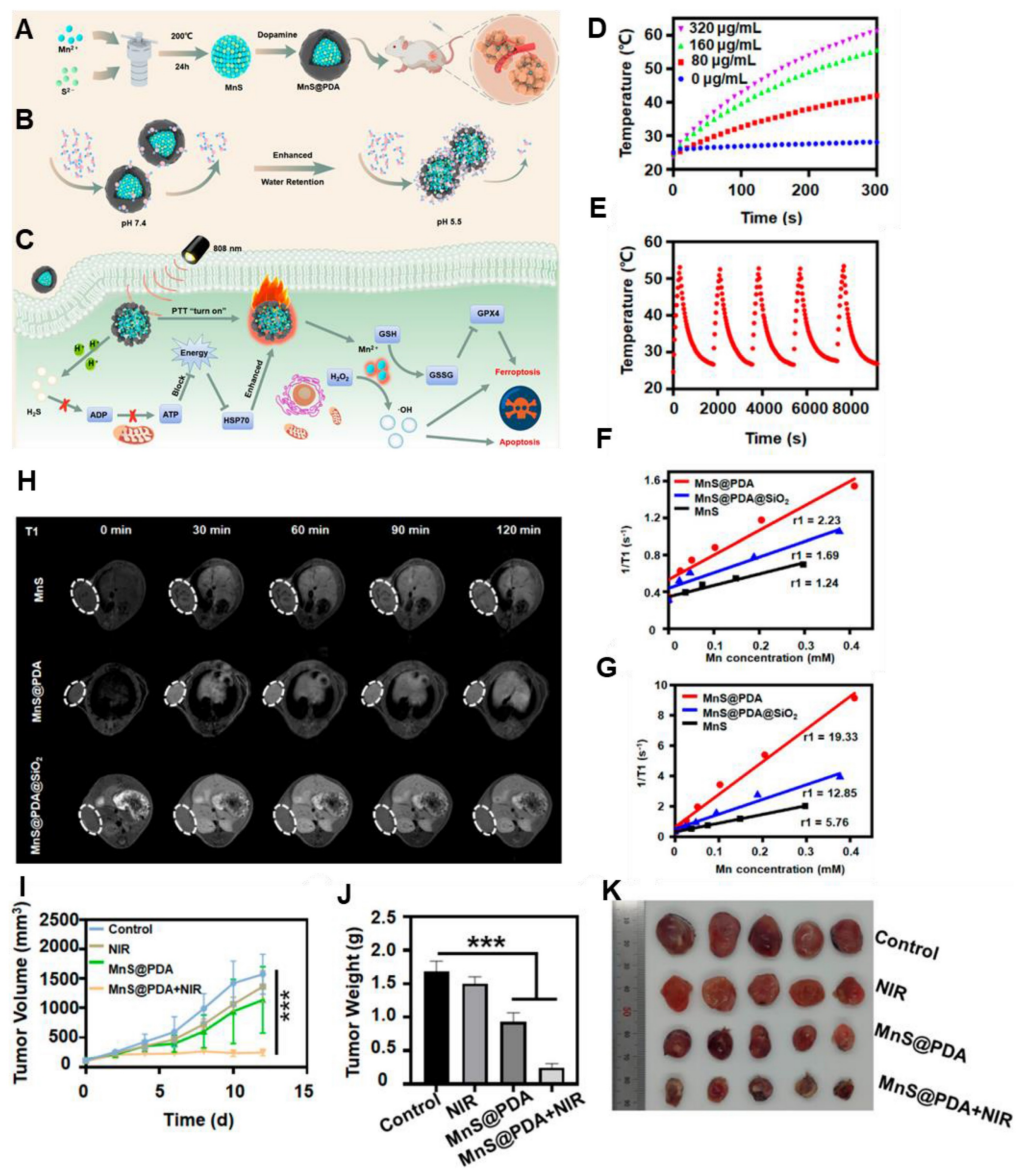
** (A)** Schematic Illustration of (A) the Detailed Synthesis mechanism of MnS@PDA, **(B)** With varying pH conditions, the mechanism of PDA Shell Enhancing Water Molecules Interaction **(C)** Mechanism of MnS@PDA for PTT and Ferroptosis Therapy of Tumors** (D)** With different concentration**,** photothermal temperature changes of MnS@PDA **(E)** Temperature changes of MnS@PDA with 5 times NIR irradiation cycle. **(F)** Longitudinal relaxation detection under pH 7.4 and **(G)** pH 5.5. **(H)** Longitudinal relaxation detection of mice at 30, 60, 90, and 120 min postinjection of various samples with an Mn dose of 3.8 mg·kg^-1^ **(I)** Tumor growth curves, **(J)** final tumor weight, and **(K)** Tumor photographs after various treatments (***P < 0.001). Adapted with permission from 78, copyright 2024 American Chemical Society.

Currently used photothermal agents like Au nanoparticles or graphene and graphene oxide sheets are not biodegradable and may have a poor pharmacokinetic profile. In this regard, Cheng *et al.* reported MRI-guided PTT using manganese carbonate nanoparticles coated with polydopamine (MnCO_3_@PDA NPs) 76. Other than BSA or PDA coating, Mn(III) porphyrins can also be used. For example, Jing *et al.* conjugated Au nanoshells with Mn(III) porphyrin 77. To increase the colloidal stability and for longer blood circulation time, PEG was covalently linked to its surface. The complex entrapped DOX to develop DOX@PLA@AuPEG-MnP nanoparticle (diameter of 123.6 nm). Under NIR light irradiation, efficient photo-hyperthermic effect and triggered release of DOX were observed. The Mn(III) porphyrin provided a relaxivity at 0.5 T of 22.18 mM^-1^ s^-1^. *In vivo* MRI relaxation was evaluated in HT-29 tumor-bearing mice and a positive MRI contrast at the tumor site was observed. Polydopamine (PDA) has also been used in this regard by several groups. It is observed that PDA cloaking can increase the pH response of the MnS nanoclusters and is effectively attributed to PTT. Furthermore, it released Mn^2+^ in a sustained manner and achieved 19.33 mM^-1^s^-1^ *T1* relaxation **(Figure 4)** 78. Besides, BSA-stabilized MnO_2_ with cross-linked indocyanine green has also been developed for MRI-guided PTT. Under NIR irradiation of 808 nm (laser power of 0.5 W cm^-2^), tumors recovered completely after 13 days of intratumoral administration 63. Moreover, a higher *T1* rate of relaxation is found (70.6 mM^-1^ s^-1^ at 0.5 T). Since 2D MnO_2_ nanosheets possess an inherent photothermal conversion capability of η: 21.4 % and can exhibit *T1*-weighted MR imaging capabilities as pH and redox response, it can be useful for inhibiting tumor growth 79.

Heat shock protein (HSP) production may be induced by heterogeneous heat distribution during photothermal treatment (PTT), which could result in heat resistance and decreased effectiveness. To improve therapeutic effects and enable on-demand drug release, Jin *et al.* created a Co-P@mSiO2@Dox-MnO_2_ theragnostic agent for pH-activated *T1/T2* MRI-guided simultaneous photothermal and chemotherapy. Furthermore, 'cold PTT' is being investigated to lessen harm to healthy tissues, and PTT in combination with other therapies can increase the effectiveness of treatment 80.

So, Mn-based contrast agents for MRI-guided photothermal therapy have the potential to improve tumor visualization and enable targeted hyperthermic therapy at the same time. They are an important tool in the development of non-invasive cancer treatments because of their capacity to produce reactive oxygen species and increase imaging accuracy, both of which boost therapeutic efficacy.

### 3.4 Mn-based contrast agents for MRI-guided sonodynamic therapy

The integration of sonodynamic therapy (SDT) with nanotechnology offers a novel approach to cancer treatment by enhancing reactive oxygen species (ROS) production in tumor cells. Nanomaterials, such as sonosensitizers, are engineered to improve ultrasonic responsiveness and target tumor tissues specifically, minimizing harm to healthy cells. This strategy increases the therapeutic efficacy of SDT while reducing side effects, leading to tumor cell death and improved cancer treatment outcomes. For instance, Huang *et al.* reviewed the anti-tumor mechanism of SDT, highlighted advancements in nanotechnology-based SDT treatments, and explored the role of nanomaterials in SDT combination therapies, paving the way for designing innovative sonosensitizers^81^.

The perovskite-type sonosensitizer manganese vanadate (MnVO₃) improves sonodynamic treatment (SDT) by generating reactive oxygen species (ROS) during ultrasound, which destroys cancer cells. While its biodegradability guarantees its safe elimination from the body, its combination with chemodynamic treatment (CDT) enhances its effectiveness by raising ROS production and decreasing glutathione in acidic tumor settings. MnVO₃ provides the possibility for safe, biodegradable cancer treatments, low systemic toxicity, and strong antitumor efficacy 82.

**Figure 5:**
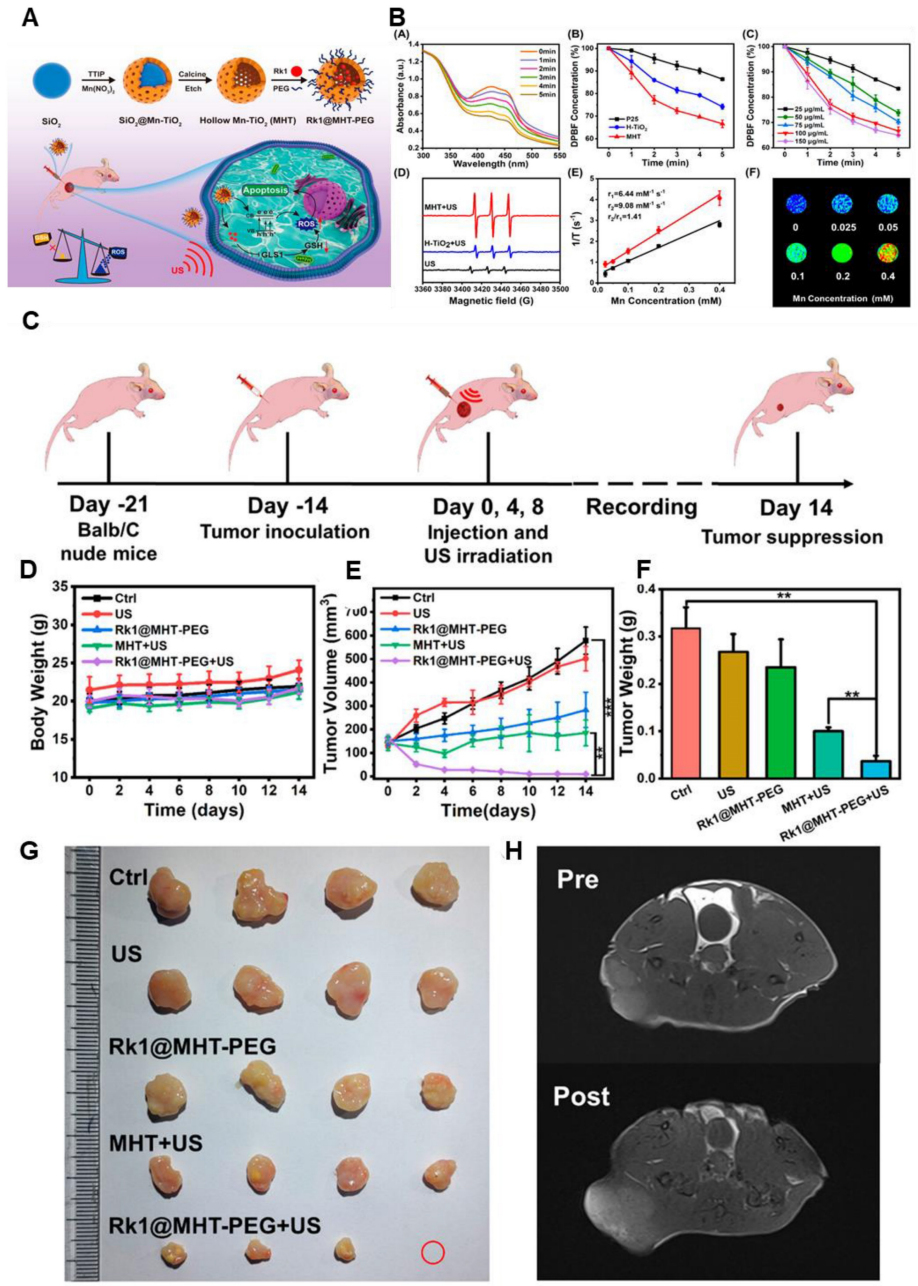
** (A)** Schematic Illustration of Rk1@MHT-PEG Preparation and Antitumor Mechanism. **(B)** A) DPBF UV-vis absorption curve in MHT aqueous solution under various ultrasonic irradiation times. B) The relative DPBF concentrations of MHT, H-TiO_2_, and P25 were exposed to ultrasound radiation, respectively. C) Relative absorption of DPBF following coexistence with varying MHT concentrations via ultrasonic irradiation. D) ESR tests of various groups with the _1_O^2^ probe TEMP. E) The MHT relaxation curve. F) MHT *T1*-weighted MR imaging at varying Mn concentrations. In the relevant groups, US irradiation (1.0 MHz, 2 W/cm^2^, 50% duty cycle) was administered. *p < 0.05, **p < 0.01, and ***p < 0.001 are the means ± SD of three separate experiments. **(C)** Diagrammatic representation of the Rk1@MHT-PEG nanoprobe animal experiment procedure. **(D-F)** Body weight, tumor weight on day 14, and tumor volume changes in mice treated for 14 days. (G) Photos of the dissected tumor were taken on day 14 by several groups. **(H)** *T1*-weighted MR imaging before and following the insertion of a nanoprobe. In the relevant groups, ultrasonic irradiation (1.0 MHz, 1.5 W/cm_2_, 50% duty cycle) was administered for 3 minutes. The means ± SD of at least three separate experiments are used to display all data. **p<0.01, ***p<0.001, *p<0.05. Adapted with permission from 83, copyright 2023 American Chemical Society.

Nonetheless, Ginsenoside Rk1-loaded manganese-doped hollow titania nanoparticles (Rk1@MHT) improve drug transport and boost ROS generation, which improves tumor sonodynamic treatment (SDT). ROS formation is catalyzed by manganese doping, and drug loading efficiency is maximized by the hollow titania structure. By increasing oxidative stress, ginsenoside Rk1 prevents the growth of cancer cells. By raising ROS levels and decreasing the bandgap of titania, the Rk1@MHT sonosensitizer increased the efficacy of SDT and eliminated tumors in mice. High-performance, noninvasive SDT-based cancer treatment appears to be possible with this strategy **(Figure 5)** 83.

An efficient template for creating metallic oxide nanoparticles, including MnO₂ (MnNPs@Keratin) and GdO₃ (GdNPs@Keratin), was shown by Yan Li *et al.* These nanoparticles show good colloidal stability, biocompatibility, and outstanding *in vitro* and *in vivo* MR imaging capabilities. The therapeutic potential of keratin is increased by its cysteine-rich composition, which imparts redox-responsive drug-release behavior 84. MRI-guided SDT can be utilized to precisely treat deep-seated resistant-pathogen infections in addition to diagnosing cancer and treating it with SDT 85. A bacterial-targeting peptide, a porphyrin sonosensitizer (MnTCPP), and a PEG shell were combined to create a polymer-peptide-porphyrin conjugate (PPPC), which allows for accurate real-time monitoring of infected areas. Nano-aggregates and increased sonosensitizer accumulation result from the PEG layer dissociating at the infection site following intravenous injection because of over-expressed gelatinase. This makes it possible for MRI-based monitoring and effective ultrasonic irradiation-based bacterial removal.

Despite its benefits—such as deep tissue penetration, non-invasiveness, and no harm to nearby organs—sonodynamic therapy (SDT) has drawbacks, including lengthier treatment times and limited penetration depth because of ultrasound attenuation. MRI guidance can help with these problems and increase the efficacy of treatment. The chosen sonosensitizer and the medical problem being treated determine the safety and effectiveness of SDT. Future developments in SDT as a treatment alternative could improve and broaden its application.

### 3.5 Mn-based contrast agents for MRI-guided surgery

The potential of manganese-based (Mn-based) contrast agents to improve tumor visibility has led to an increase in their use in MRI-guided surgery. They have several advantages such as their lower toxicity, higher bio-compatibility when used in a particular amount. Not only that, it has a natural affinity for cellular transport mechanisms, such as calcium channels, which enables targeted imaging, further improving surgical accuracy. Their paramagnetic characteristics enhance *T1*-weighted imaging, facilitating accurate surgical planning and tumor localisation. Additionally, Mn-based medicines lower the risk of surgery by helping to define the boundaries of the tumor and the surrounding tissues. By guaranteeing more precise, targeted tumor removal during treatments, these medicines present a promising strategy for enhancing surgical outcomes and have the possibility for real-time monitoring. MRI-guided surgery refers to surgical procedures in which magnetic resonance imaging (MRI) technology provides real-time, high-resolution images that guide the surgeon during the operation. This approach offers several advantages in terms of precision, visualization, and the ability to navigate through complex anatomical structures 86. MRI-guided surgery is commonly employed in various medical fields, including neurosurgery, orthopedic surgery, and certain cancer treatments 87. It enables surgeons to make informed decisions and enhance the accuracy of their procedures, ultimately improving patient outcomes. It has recently received enormous attention owing to its specificity and safety. Especially, MRI imaging has been proven to have multiple advantages in clinics. It is observed that Mn^2+^-based MRI CAs are capable of being used in such applications.

**Figure 6:**
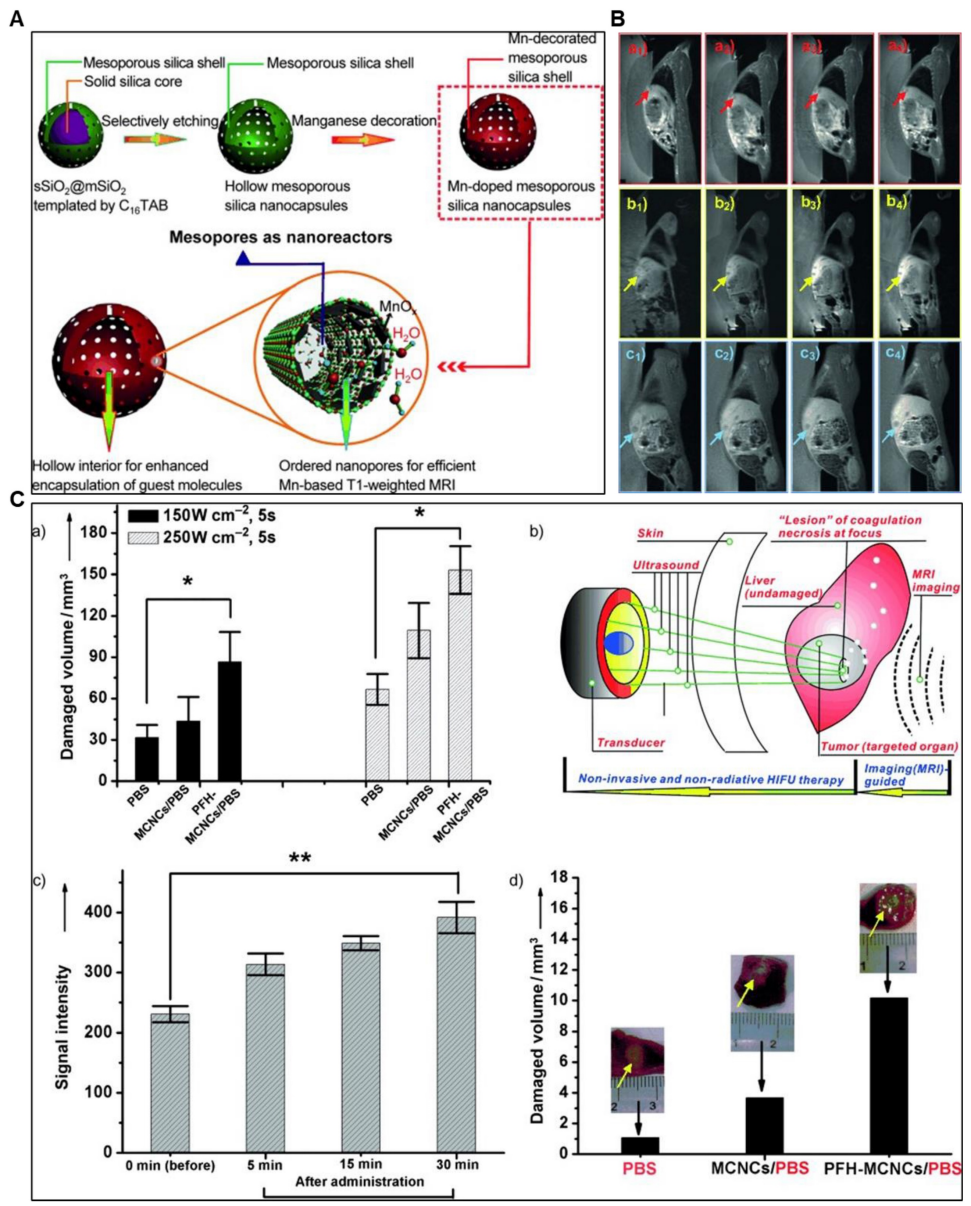
** (A)** Schematic illustration of the synthetic protocol for MCNCs and their corresponding microstructures. **(B)** *In vivo T1*-weighted MRI of rabbits with VX2 liver tumors before (a1, b1, c1) and after agent administration at 5 min (a2, b2, c2), 15 min (a3, b3, c3), and 30 min (a4, b4, c4) via the ear vein. Agents: PBS (a1-a4), MCNCs/PBS (b1-b4), PFH-MCNCs/PBS (c1-c4). Arrows mark the tumor. PBS = phosphate-buffered saline. **(C)** a) Coagulated-tissue volume of degassed bovine liver was analyzed after injecting PBS, MCNCs/PBS, or PFH-MCNCs/PBS (200 μL each) under 150 W/cm² or 250 W/cm² irradiation for 5 seconds (*P < 0.05). b) MRI-guided HIFU enables precise surgery for hepatic neoplasms in rabbits by targeting tumors under real-time imaging. c) *T1*-weighted MRI signal intensities of tumors significantly increased after intravenous PFH-MCNCs/PBS administration (**P < 0.005). d) Coagulated necrotic tumor volume was measured after MRI-guided HIFU exposure (150 W/cm², 5 s) in rabbit liver tumors treated with different agents (inset: tumor photos post-HIFU). Copyright (2023) with permission from the American Heart Association Adapted with permission from^88^, copyright 2011, Wiley-VCH.

Chen *et al.* synthesized manganese-based multifunctional mesoporous composite nanocapsules (MCNCs) as contrast agents (CAs) and synergistic agents (SAs) for MRI-guided High-Intensity Focused Ultrasound (HIFU) cancer surgery. The MCNCs' unique structure offers key advantages: a paramagnetic mesoporous shell with confined manganese oxide for efficient *T1*-weighted MRI and hollow interiors for PFH delivery, enhancing HIFU synergy. In rabbit liver tumors, MCNCs enabled precise ultrasound targeting and improved therapeutic outcomes. These nanostructures show potential for delivering molecules like chemotherapeutics, paving the way for MRI-based diagnosis, thermal chemotherapy, and imaging-guided HIFU cancer surgery **(Figure 6)** 88.

Notably, MRI has already been successfully established as an imaging modality in clinics. Therefore, using this technique in imaging-guided surgery would incur nominal modifications and costs. However, extensive research may be needed to enhance rationality and reduce the secondary toxicity of imaging contrast agents. Herein, manganese complexes can play a crucial role shortly.

Nonetheless, by improving imaging contrast, Mn-based contrast agents for MRI-guided surgery provide several benefits, including enhanced tumor visualization. They are a promising tool for accurate, minimally invasive cancer therapies, improving surgical outcomes and therapeutic efficacy because of their capacity to offer real-time monitoring, lower surgical risks, and enhance tumor targeting.

### 3.6 Mn-based contrast agents for MRI-guided radiotherapy

Manganese-based (Mn-based) contrast agents are becoming cutting-edge instruments for MRI-guided radiation therapy, fusing therapeutic enhancement with accurate imaging. By improving *T1*-weighted MRI, their paramagnetic qualities allow for precise tumor localization and treatment planning. Additionally, by upsetting hypoxia and encouraging the production of reactive oxygen species (ROS), Mn ions alter the tumor microenvironment and improve radiosensitivity. Because of their dual purpose, Mn-based medicines are bright prospects for increasing the accuracy and effectiveness of radiation therapy and enhancing the results of cancer treatment.

Radiotherapy is necessary for about half of cancer patients. It uses high-energy X-rays or γ-rays to create ROS, which damages DNA, triggers apoptosis, and destroys tumors. However, the effectiveness of radiation is limited by hypoxic tumor microenvironments (TME) in solid tumors. This is addressed by manganese-based nanomaterials, especially manganese oxides, which increase the body's natural generation of oxygen. Dose optimization and better radiation results are made possible by MRI-guided imaging, which guarantees ideal pharmacokinetics, pharmacodynamics, and accurate accumulation of contrast agents 89.

**Figure 7:**
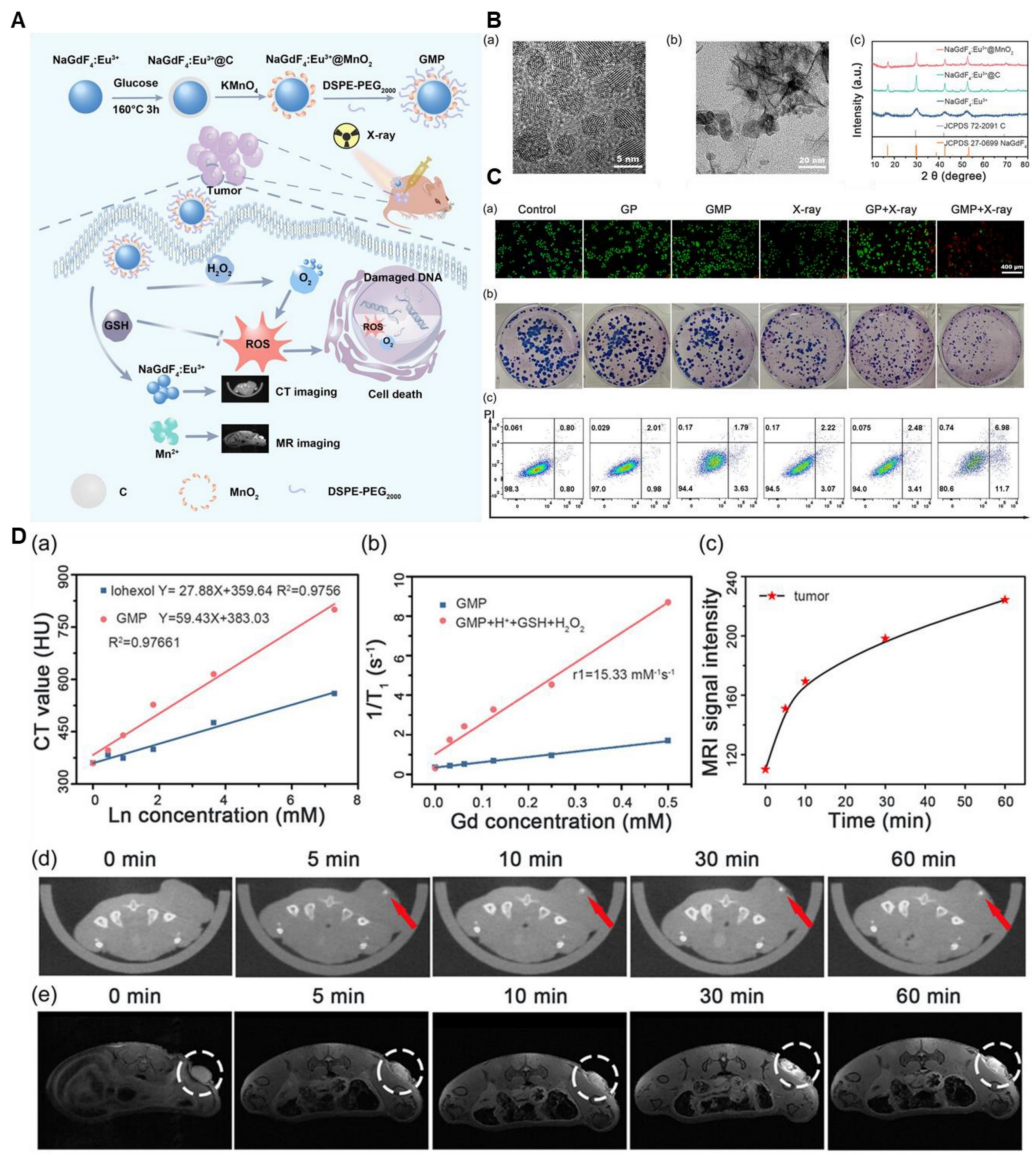
**(A)** Schematic diagram of the MRI/CT guided radiotherapy. **(B)** TEM (a,b) and XRD of NaGdF_4_:Eu^3+^ and NaGdF_4_:Eu^3+^@MnO_2_ nanoparticles. **(C)** (a) Fluorescence images of HeLa cells with different treatments after the live/death staining by Calcein and PI. PI stained the necrotic cells with red fluorescence and Calcein stained the live cells with green fluorescence. (b) Colony formation assay of HeLa cells under different treatments. (c) Flow cytometric analysis of HeLa cells apoptosis with different treatments. Apoptotic ratio: a sum of the early apoptotic ratio in the Q3 region and the late apoptotic ratio in the Q2 region. The dose of X-ray was 6 Gy. **(D)** a) In vitro CT values of GMP NP aqueous solutions with different concentrations of Ln (Gd + Eu). b) The longitudinal relativities (*r1*) of GMP NPs in different solutions. c) The corresponding signal intensity of MR in tumor after intratumor injection of GMP NPs solution at different times. d) *In vivo* CT images and e) MR images of HeLa-tumor-bearing nude mice at 0, 5, 10, 30, and 60 min after intratumor injecting GMP NPs solution (4 mg mL^-1^, 20 μL). The circled part was the tumor. Adapted with permission from^91^, copyright 2024 Wiley-VCH.

For instance, a review by Cai *et al.* emphasizes manganese oxide (MON) nanoparticles (NPs) as potentially useful tools for MRI-based theranostics. The study discusses their use as MRI contrast agents for tumor diagnosis and detection as well as how they may improve the effectiveness of chemotherapy and radiation therapy by tackling issues with the tumor microenvironment. The uses of MONs in photothermal and photodynamic therapy are also examined, demonstrating their adaptability in transforming the treatment of cancer. According to the results, MONs are a versatile tool for enhancing cancer treatment and detection 90. Moreover, researchers have played a significant role in advancing intelligent drug delivery systems based on MnO_2_. Among these breakthroughs, Song and colleagues detailed the application of MnO_2_ nanoparticles loaded with Doxorubicin (Dox), modified with hyaluronic acid (HA), and conjugated with mannan (referred to as Man-HA-MnO2 NPs). This innovative approach aimed at targeting 4*T1* mouse breast cancer cells, enhancing chemotherapy, and enabling imaging. Moreover, Li and his team successfully reverse radiotherapy-resistant factors for radiotherapy sensitization and MRI **(Figure 7)**. They integrated Gd inside the Mn-complex to attain CT imaging for 3D visualization. It also enhanced the radiotherapeutic efficacy 91.

Tumor hypoxia is addressed by tumor-associated macrophages (TAMs) in hypoxic tumor locations, in conjunction with MnO_2_ nanoparticles (NPs) that react with hydrogen peroxide (H_2_O_2_) to produce oxygen (O_2_) and control pH. In addition to targeting tumor cells, hyaluronic acid (HA) also transforms anti-inflammatory M_2_ TAMs into pro-inflammatory M1 macrophages, increasing the effectiveness of nanoparticles and overcoming chemoresistance. Mn^2+^ ions are released when Man-HA-MnO_2_ NPs react with H_2_O_2_, which enhances tumor imaging and *T1/T2*-weighted MRI detection capabilities. 92 MRI agents based on hyaluronic acid (HA) present several advantages, including their biocompatibility, ability to target specific tissues or cells, biodegradability, and enhanced retention 93. However, they have limitations, such as potential issues with relaxivity, stability, synthesis complexity, size constraints, potential immunogenicity, and variability. The choice to employ HA-based MRI agents depends on the precise imaging goals and the meticulous assessment of these pros and cons, tailored to the unique requirements of the given application 94.

Similarly, Liu and colleagues created a dye-based MnO_2_ nanoparticle (NP) system for MRI-guided radiation. MnO_2_ nanoparticles were effectively loaded with the radiosensitizer acridine orange (AO), which was then released under regulated conditions. By increasing DNA damage, the device produced oxygen (O_2_) inside cells, improving the results of radiation. Furthermore, MnO_2_ nanoparticles break down in acidic settings, releasing Mn^2+^ for MRI monitoring. According to the study, mMnO_2_ presents a suitable platform for the development of composite radiosensitizers for use in radiation treatment 95.

MRI-guided radiotherapy makes Adaptive treatment planning possible, which permits real-time modifications for the best possible radiation delivery. Manganese complexes, especially nanoparticles (NPs), are now essential for MRI-guided radiation therapy because they can efficiently target a variety of malignancies. To investigate their potential for boosting theranostic properties and boosting therapeutic efficacy, more study is required on other Mn-complexes. Hence, for MRI-guided radiotherapy, Mn-based contrast agents provide improved tumor imaging and treatment accuracy. Mn compounds successfully combine diagnostic and therapeutic roles by enhancing MRI contrast, enabling regulated drug delivery, and intensifying oxidative stress through catalytic processes, increasing radiation outcomes with fewer side effects and greater efficacy.

### 3.7 Mn-based contrast agents for MRI-guided photodynamic therapy

In MRI-guided photodynamic treatment (PDT), manganese-based (Mn-based) contrast agents are becoming more and more popular as dual-purpose materials. Because of the paramagnetic nature of Mn ions, these medicines offer improved *T1*-weighted MRI, allowing for accurate tumor monitoring and localization. By producing reactive oxygen species (ROS) in response to light activation, they serve as catalytic agents in PDT and efficiently target cancer cells 96. The stability, biocompatibility, and therapeutic effectiveness of Mn-based medicines are improved when they are combined with nanocarriers or photosensitizers (PS). Furthermore, Mn ions help modulate the tumor microenvironment by interfering with hypoxia, which enhances PDT results. Mn-based contrast agents are useful in advanced cancer theranostics because of their multifunctionality, which seamlessly bridges imaging and treatment by combining diagnostic and therapeutic properties.

**Figure 8:**
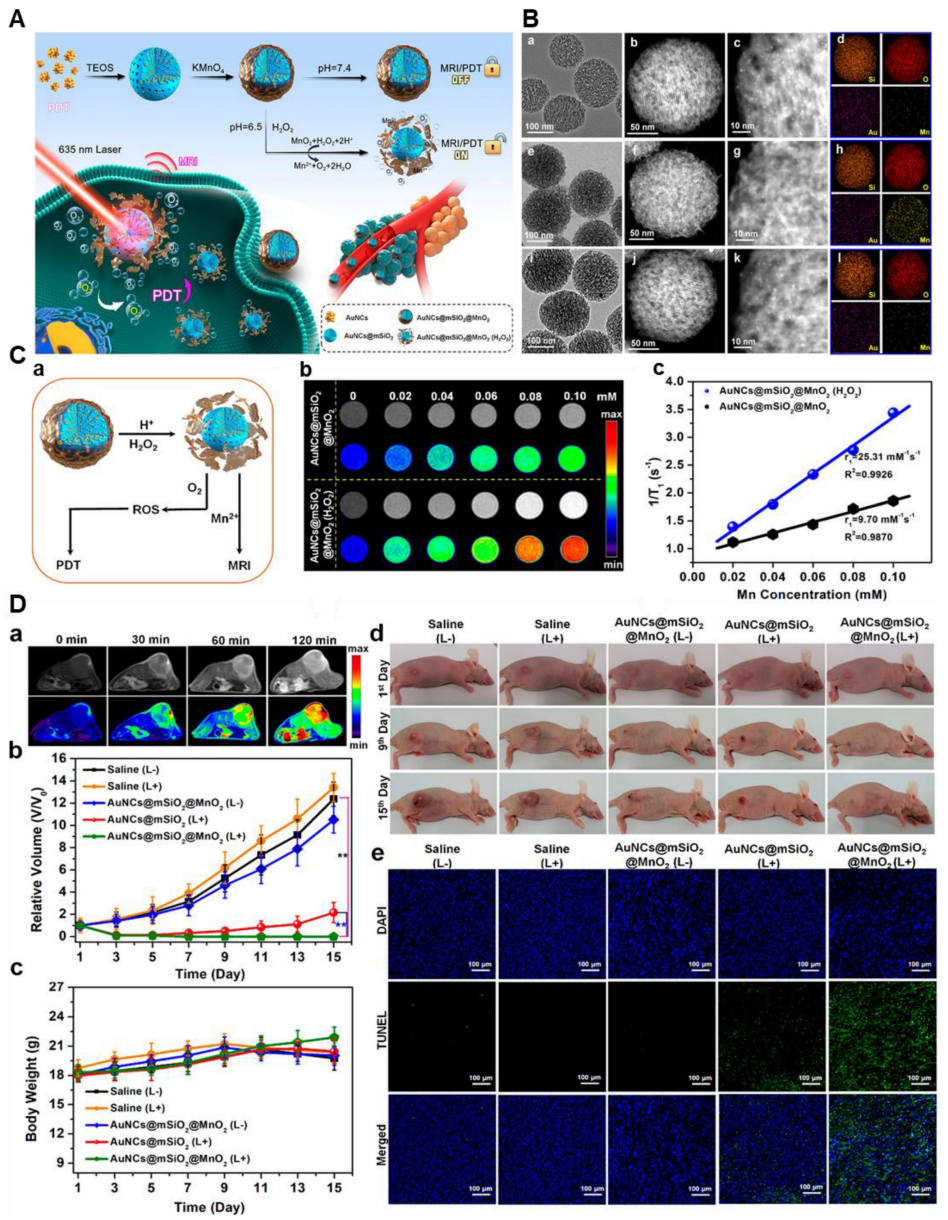
** (A)** AuNCs@mSiO_2_@MnO_2_ nanozyme synthesis process for H_2_O_2_-responsive "Off/On" regulation and upgrade of MR imaging and photodynamic therapy. **(B)** TEM, STEM, high-amplification STEM, and component planning pictures of the synthesized samples. (a-d) AuNCs@mSiO_2_; (e-h) AuNCs@mSiO_2_@MnO_2_; and (I-l) AuNCs@mSiO_2_ @MnO_2_ (H_2_O_2_). **(C)** H_2_O_2_ -responsive MR, O_2_, and ^1^O_2_ age execution. (a) "Off/on" tweak and upgrade of MR imaging, PDT; (c) *T1* - weighted MR imaging; (d) longitudinal MR unwinding bends of samples. **(D)** *In vivo* MR imaging and PDT. (a) *T1* - *T1*-weighted MR imaging of mice infused intravenously with the sample. (b) growth volume change bends of various gatherings; (c) body weight change bends of various gatherings. (d) photos of mice in various gatherings; and (e) DAPI and TUNEL staining pictures of MDA-MB-435 growths in various gatherings. Adapted with permission from 99, copyright 2021, the American Chemical Society.

Photodynamic therapy (PDT) is a biomedical procedure that employs a photosensitizing substance and particular light wavelengths to specifically eliminate or harm designated cells, including cancerous cells or irregular tissue. PDT entails the introduction of a photosensitizer into the desired region, where it accumulates and is subsequently subjected to light of the suitable wavelength 97. Through the combination of photosensitizers (PS), oxygen, and light, photodynamic treatment (PDT) produces reactive oxygen species (ROS), which leads to necrosis or apoptosis and necrobiosis. It is extensively used in ophthalmology, dermatology, and cancer treatment. Theranostic properties of PS formulations are improved by advancements, which allow for precision imaging-guided PDT by triggering diagnostic signals in response to stimuli unique to tumors. To provide optimal treatment planning and better results, PDT relies on precise dosimetry and monitoring, taking into account tumor features, PS concentration, location, light distribution, and oxygen levels. Additionally, it involves post-treatment assessment to gauge the response, potential recurrence, and alleviation of symptoms **(Figure 8)** 99. This setup effectively suppressed singlet oxygen (^1^O_2_) production, resulting in the "off" state for both PDT and MR imaging due to the stable MnO_2_ shield. Nevertheless, in the acidic tumor microenvironment, the MnO_2_ case underwent a reaction with H_2_O_2_, causing its degradation and activating both MR imaging and photodynamic therapy (PDT) concurrently. The MR imaging demonstrated a longitudinal relaxation rate of 25.31 mM^-1^ s^-1^, ensuring a 74% yield of singlet oxygen (^1^O_2_) owing to the sufficient generation of oxygen (O_2_) 99-100.

Though, photodynamic therapy (PDT) offers localized, non-ionizing treatment with reduced radiation risks but faces challenges like limited penetration depth and variable outcomes based on photosensitizers and patient factors. MRI-guided PDT enhances efficacy by refining treatment precision. Combining PDT with other modalities may further improve effectiveness while maintaining minimal invasiveness.

Apart from metal nanoparticles and nanoclusters, scientists nowadays are working on biodegradable theranostic probes for this purpose. Such initiatives may be crucial for designing new-age theranostic probes having multiple activation-induced imaging capabilities.

Therefore, Mn-based contrast agents improve MRI-guided photodynamic therapy by producing reactive oxygen species (ROS), enhancing tumor imaging, and enabling targeted therapy. Their simultaneous role in treatment and imaging, along with the catalytic qualities of Mn, boosts therapeutic efficacy and presents a viable strategy for accurate, non-invasive cancer treatment.

### 3.8 Mn-based contrast agents for MRI-guided immunotherapy

Manganese-based (Mn-based) contrast agents combine therapeutic advantages with diagnostic imaging to improve MRI-guided immunotherapy. Their paramagnetic characteristics enhance *T1*-weighted imaging, allowing for accurate tumor monitoring and localization. Mn ions also alter the tumor microenvironment by promoting immune cell infiltration and upsetting hypoxia. Because of their dual purpose, Mn-based medicines can effectively combine immunotherapy with MRI diagnostics, providing a prospective means of enhancing the effectiveness of treatment for immune-related disorders and cancer.

The immunomodulatory qualities of manganese (Mn) are used in manganese-based (Mn-based) tumor immunotherapy to strengthen the body's anti-cancer defenses. Mn ions improve tumor identification and removal by activating immune cells. By delivering immune-stimulating chemicals, Mn-based nanoparticles encourage the generation of cytokines and the entry of immune cells into tumors. Improvements in Mn-based nanoplatforms for imaging-guided methods, immunotherapy, and multimodal synergistic medicines are highlighted by Zhang *et al.* The study highlights Mn's potential to improve cancer immunotherapy outcomes by addressing its characteristics, therapeutic potential, and obstacles 101.

Cyclic dinucleotides (CDNs) are a class of molecules made up of two nucleotides combined in a cyclic structure. They participate in cellular signaling, specifically in the activation of the STING (stimulator of interferon genes) pathway, which is essential for triggering immune responses, particularly in the context of cancer and viral infections. Cyclic dinucleotides, like cyclic GMP-AMP (cGAMP), are used as agonists to stimulate the STING pathway, which improves immune system activation against tumors and pathogens. This STING pathway is activated by cyclic dinucleotide-manganese particles (CDN-Mn), which also increase immunological defense by producing type I interferon and cytokines. Cyclic dinucleotides are delivered to immunological and tumor cells by CDN-Mn particles, which enhance immune cell infiltration and initiate systemic reactions. Using Mn²⁺ and CDN STING agonists, Sun *et al.* created a metalloimmunotherapy prototype that produced the self-assembling nanoparticle CDN-Mn²⁺ (CMP). Strong anti-tumor immunity and great therapeutic efficacy with modest STING agonist dosages were shown by CMP in animal models, enhancing delivery and efficacy 102. In the tumor microenvironment, the cGAS-STING pathway is activated by the ATP-responsive manganese-based bacterial material (*E. coli*@PDMC-PEG). Elevated ATP levels cause the material to break down, producing Mn^2+^ ions that increase the sensitivity of the cGAS enzyme to bacterial extracellular DNA, thereby activating the pathway in concert. According to *in vivo* research, *E. coli*@PDMC-PEG and VNP20009@PDMC-PEG successfully prevented the formation of liver cancer in rabbits and melanoma in mice, indicating their potential as therapeutic agents 103. Subsequently, A manganese-based nanoplatform (MPCZ NPs) that triggers the cGAS-STING pathway was created by He and his associates. When subjected to a NIR laser and elevated endogenous GSH levels, MPCZ NPs, which coat ZPP on hMnO_2_ NPs and load PDA with NH4HCO3, release Mn²⁺ in tumor cells. This increases innate immunity by inducing the creation of ROS and strengthening the STING pathway. MPCZ NPs successfully suppressed tumor growth in the 4*T1* tumor-bearing mouse model, demonstrating their potential to improve anticancer immunotherapy 104. Consequently, Li *et al.* conjugated biocompatible cationic biopolymers with cGAMP to generate nanocomplexes that were affixed to APC-targeting microbubbles (MBs), thereby developing a platform for ultrasound (US)-guided cancer immunotherapy. By delivering cGAMP into the cytosol, sonoporation primed antigen-specific T cells by triggering proinflammatory and cGAS-STING pathways 105. Simultaneously, Cai *et al.* developed a strategy using a bispecific antibody (BsAb) with a manganese oxide-doped silicate nanosystem to deliver minicircle DNA. It activated the cGAS-STING pathway, redirected host lymphoid cells to target cancer, and achieved a high rate of antibody discharge over five days, illustrating manganese's indirect role in supporting immune functions for cancer therapy 106.

**Figure 9:**
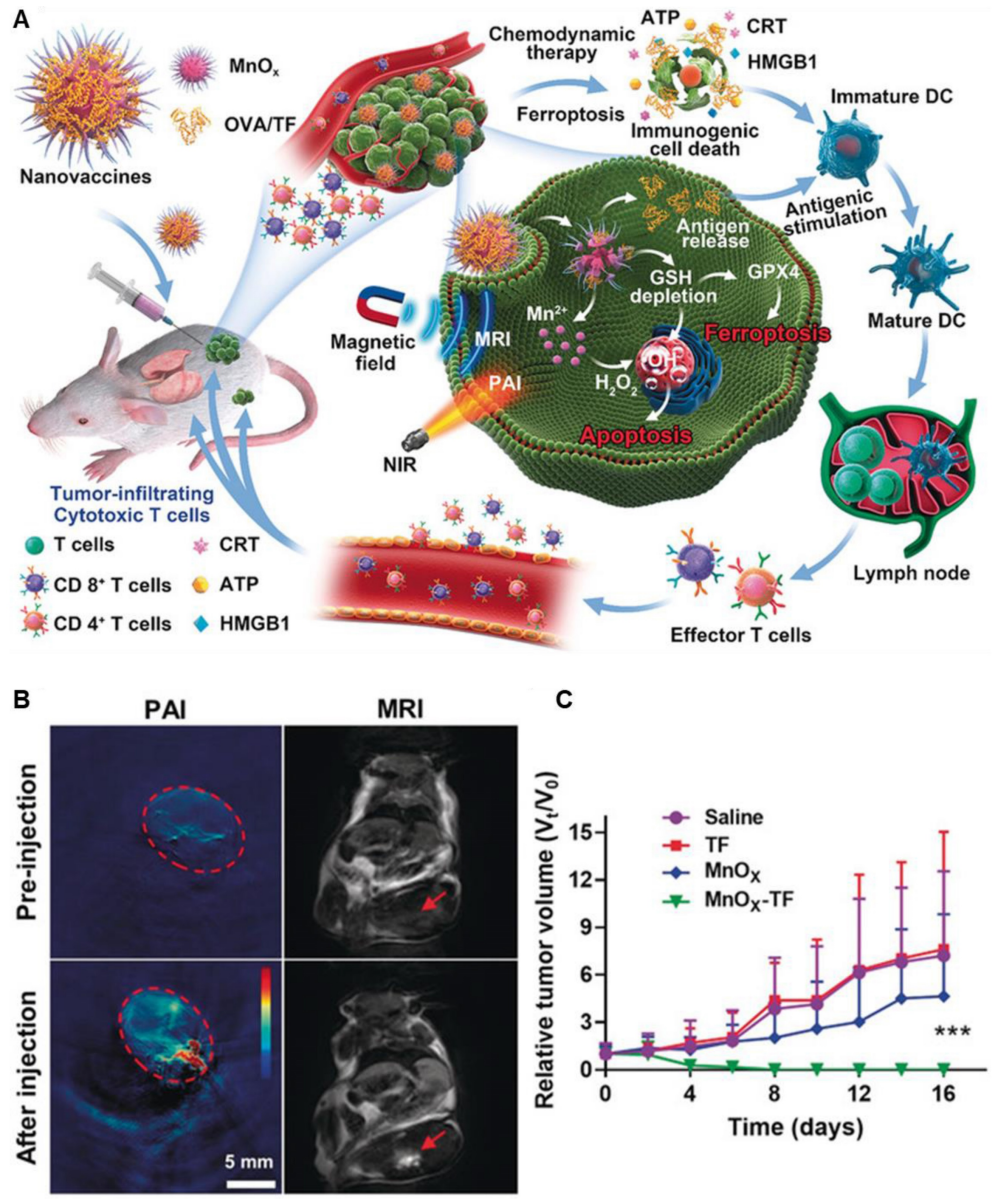
** (A)** Illustration of MnOx-OVA/tumor cell fragment (TF) nanovaccines for MR/PA dual-mode imaging-induced cancer immunotherapy. **(B)** In vivo MR/PA dual-mode imaging. **(C)** Relative tumor-growth curves of distant tumors. Adapted with permission from^107^, copyright 2020, Wiley-VCH GmbH.

Moreover, manganese-based nanoparticles are regarded as biocompatible and exhibit diverse applications in the field of nanomedicine. Sun *et al.* used such a biodegradable Mn-based theranostic agent for MRI-guided dual immuno-chemodynamic combination therapy. In reality, they developed MnO nanoparticles (NPs) coated with hollow mesoporous silica and further modified them with the tumor-homing peptide iRGD (CRGDKGPD). The resulting NPs, termed MnO@mSiO_2_-iRGD NPs, were utilized in an MRI-guided tumor immuno-chemodynamic combinatory therapy. These NPs offered multiple benefits, including activation of the cGAS-STING pathway for immunotherapy, upregulation of reactive oxygen species through Fenton-like reactions, and *T1*-weighted MRI capabilities 106. Additionally, Ding *et al.* synthesized Manganese oxide nanospikes (MnOx NSs) which are responsible for the tumor microenvironment (TME)-responsive nano adjuvants and immunogenic cell death (ICD) drugs for cancer nanovaccine immunotherapy. With high antigen-loading capacity, they combine ICD-based chemodynamic therapy, ferroptosis induction, and antigen stimulation, offering synergistic immunopotentiation, dual-mode imaging (MRI/photoacoustic), and effective inhibition of tumor growth and metastasis **(Figure 9)** 107.

Manganese is essential for immunological function, but too much of it can be harmful. If properly optimized, MRI-guided immunotherapy presents a viable clinical alternative to maintaining a balanced intake. Nevertheless, Mn-based contrast agents improve tumor targeting and immune response, which improves MRI-guided immunotherapy. They are potential prospects for clinical cancer treatment applications because of their capacity to strike a balance between safety and efficacy.

### 3.9. Mn-based contrast agents for MRI-guided combinatory therapy

Multimodal/synergistic/combinatory therapy has become a dominant choice owing to its particular and targeted approach toward the therapeutic modality 108,109. In a combinatory setup, two or more different therapeutic regimens must be introduced simultaneously for higher efficacy. Often a part of these therapeutic modalities takes quick action against the disease, whereas the other modalities support the previous one by maintaining a low and continuous therapeutic profile. For instance, photothermal therapy (PTT) is a quick and effective therapy against tumors, while other therapies like chemodynamic therapy or drug delivery act as supportive systems with PTT in a combinatory setup 73,110. There are a lot of combinatory therapies accessible. Herein, we highlight a few examples to illustrate their diverse applications. To make Mn-based combinatory theranostic systems, scientists have followed several strategies. Initially, they started with complex nanosystems and nanocomposites containing Mn ions to achieve multimodal theranostics. For example, Zhu and colleagues have developed a nanoplatform for cancer theranostics comprising manganese-loaded Gox (Glucose oxidase), paclitaxel (PTX), and a near-infrared (NIR) fluorescent dye. This nanoplatform exhibited pH-dependent behavior, resulting in the release of both manganese ions and therapeutic payloads specifically within tumor cells. Through in vitro assessments and cellular experiments, it was observed that NanoMn-GOx-PTX catalyzed the conversion of glucose into reactive oxygen species (ROS) through a cascade Fenton-like reaction, concurrently releasing PTX. The combined effects of glucose depletion, ROS generation, and the therapeutic action of PTX synergistically induced increased cytotoxicity and apoptosis in 4*T1* cancer cells 111.

**Figure 10:**
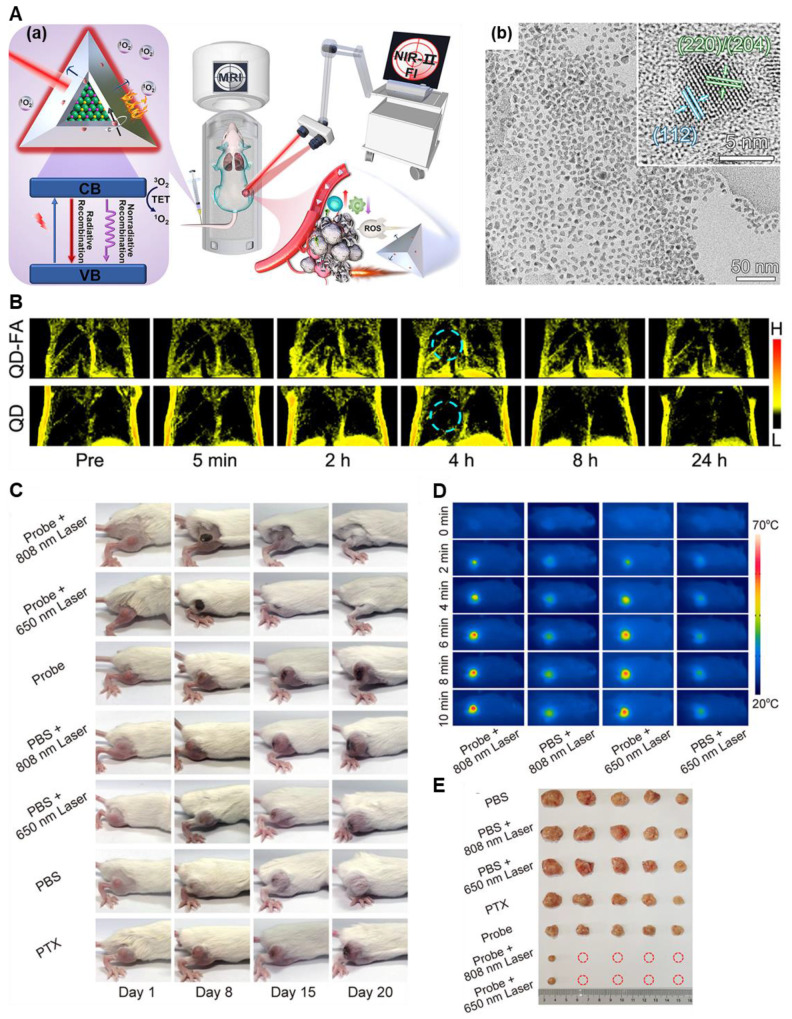
**(A) (a)** Scheme of a CISe@ZnS: Mn QD) manufactured having a lot of functionality. **(b)** structure obtained by the TEM image of the quantum dot. **(B)** lung metastasis tumor detection through *T1*-weighted MR imaging after the IV of the QD-FA probes. *In vivo,* NIR-II FI of hypodermal tumors was taken at variable time points after the IV injection. **(C)** Sample images of tumor-bearing mice undergoing the various treatments as shown. **(D)** Thermal images of mice having 4*T1* tumors injected with PBS or the QD-FA probe were recorded at different irradiation times with 808 nm laser (1.0 W·cm^-2^, 10 min) and 650 nm laser (1.0 W·cm^-2^, 10 min). **(E)** Images of 4*T1* tumors from each group were collected 20 days after treatment. Adapted with permission from 112, copyright 2022, the American Chemical Society.

Notably, when MRI has been used with other imaging modalities, the efficacy of the theranostic regimen is enhanced. In very recent work, Li *et al.* developed such a theranostic system consisting of CuInSe_2_@ZnS: Mn quantum dots (QDs). This probe can accurately detect the localization of small metastases by virtue of MRI/ NIR-II dual-modal imaging techniques and ablate the tumor by combined PDT/PTT/IT modalities **(Figure 10)** 112. This multimodal approach increased the detection efficiency up to 31.2 % and prevented tumor regrowth in 80% of mice. Such works are crucial in determining the tumor proximities and they will provide safe and non-recurring ablation of the tumor growth under the imaging guidance.

A potent cancer treatment strategy is provided by immunotherapy in conjunction with MRI-guided sonodynamic therapy (SDT). With the use of targeted SDT, which uses ultrasound to activate sonosensitizers that produce ROS and cause tumor cell death, precise tumor localization, and monitoring are made possible by MRI. Concurrently, SDT increases the immunogenicity of the tumor, increasing its vulnerability to an immune response. This dual approach, when combined with immunotherapy, enhances the body's immune response against cancer, improving therapeutic outcomes by precisely targeting the main tumor as well as any metastases. For instance, Tian *et al.* created a phenolic nanoadjuvant through metal-phenolic coordination, which allowed the sonosensitizer polymer (PEG-b-IR), GSH inhibitor (sabutoclax), Mn^2+^, and TME acidity-sensitive phenolic polymer (PEG-b-Pho) to self-assemble. The activation of the cGAS-STING pathway was accelerated by the combined action of Mn^2+^ and SDT-mediated ICD effect, which greatly increased the maturation of DCs (dendritic cells). Moreover, this phenolic nano adjuvant significantly increases the susceptibility of the tumors to PD-L1 checkpoint blockade immunotherapy, which effectively prevents lung metastasis and delays the growth of distant tumors 113. Consequently, the therapeutic constraint of inadequate antitumor immunity for improved cancer immunotherapy may be addressed by this approach.

By precisely locating tumors and continuously monitoring therapy, MRI-guided chemotherapy, and immunotherapy offer a highly tailored approach to cancer treatment. Cancer cells are destroyed by cytotoxic chemicals delivered by chemotherapy, and tumors are better recognized and destroyed by the immune system when immunotherapy is added. When drugs are delivered accurately with MRI guidance, the likelihood of side effects is reduced and the effectiveness of treatment is increased. This combined method offers a synergistic strategy for more precisely treating metastases as well as locally located tumors by stimulating the immune system in addition to actively attacking the tumor. For instance, small-sized telodendrimer and Mn^2+^-based nanodriver (PLHM) were designed that efficiently target lymph nodes via blood circulation and show tumor-preventive effects at low Mn^2+^ doses (3.7 mg kg^-1^). However, through triggering *in vivo* innate immune responses, the PLHM nanodriver also shows apparent anticancer effects in mice bearing GBM. Because cytoplasmic DNA and Mn^2+^ synergistically potentiate the efficacy of the STING pathway, PLHM and doxorubicin nanoparticles (PLHM-DOX NPs) provide superior tumor growth inhibition in mice bearing GBM. These results show that PLHM-DOX NPs can successfully induce innate immunity, boost maturation of dendritic cells, and coordinate the cascaded infiltration of CD8 cytotoxic T lymphocytes into low-immunogenicity glioblastomas. By triggering the STING pathway, these nanodivers chelated with Mn^2+^ exhibit encouraging potential for tumor prevention and anticancer effects on glioblastoma 114.

Luo *et al.* created a pH-responsive nanomodulator by co-loading MnO_2_, CaCO_3_, and curcumin (CU), a Ca^2+^ enhancer, into nanoparticles that were covered in a cancer cell membrane. The goal of this nanoplatform was to use ion fluctuation to reprogram the tumor microenvironment (TME) and provide an anticancer treatment. The resultant nanoplatform, known as CM NPs, could produce Ca^2+^ and release CU, raising Ca^2+^ levels and encouraging ROS formation in the mitochondria and endoplasmic reticulum, ultimately leading to immunogenic cell death. They could also neutralize protons by breaking down CaCO_3_ and reducing cellular acidity. To alleviate hypoxia and improve cGAS sensitivity, Mn^2+^ may break down endogenous H_2_O_2_ into O_2_, triggering the cGAS-STING signaling pathway 115. A biomimetic mineralization technique is suggested to strengthen RT-induced systemic antitumor immune responses by easily synthesizing MnO_2_ nanoparticles with high anti-programmed death ligand 1 (αPDL1) encapsulation efficiency (αPDL1@MnO_2_). By reprogramming the immunosuppressive tumor microenvironment (TME) and overcoming hypoxia-induced radio-resistance, therapeutic nanoplatforms-mediated RT can successfully elicit ICD and considerably increase tumor cell death. Additionally, under an acidic tumor pH, the produced Mn^2+^ ions from αPDL1@MnO_2_ can activate the cyclic GMP-AMP synthase (cGAS)-stimulator of interferon genes (STING) pathway, which helps to promote the maturation of dendritic cells (DCs). When αPDL1 was released from αPDL1@MnO_2_ nanoparticles, it stimulates systemic antitumor responses and intratumoral infiltration of cytotoxic T lymphocytes (CTLs), which has a potent abscopal impact that effectively prevents tumor metastasis. Overall, the biomineralized MnO_2_-based nanoplatforms are promising for improved RT immunotherapy because they provide a straightforward method for TME regulation and immune activation 116. The production of an immunostimulatory MOF (ISAMn-MOF) based on manganese ion (Mn^2+^) for cancer metalloimmunotherapy was suggested using a facile and environmentally friendly technique. In bone marrow-derived dendritic cells (BMDCs), ISAMn-MOF dramatically accelerates the activation of signaling pathways and genes linked to cyclic GMP-AMP synthase-stimulator of interferon (cGAS-STING). Compared to BMDCs treated with comparable MnCl_2_, ISAMn-MOF-treated cells release 4-fold more type I interferon and 2- to 16-fold more proinflammatory cytokines. Either by itself or in conjunction with immune checkpoint antibodies, ISAMn-MOF dramatically inhibits tumor growth and metastasis while extending the longevity of mice. Mechanistic investigations reveal that the administration of ISAMn-MOF promotes the infiltration of immune cells that stimulate tumor growth and lymphoid organs. This research sheds light on how to create bioactive MOFs for more effective cancer metalloimmunotherapy 117. In a similar manner, Zhou *et al.* reported on manganese-enriched zinc peroxide nanoparticles (MONPs) that are responsive to the tumor microenvironment (TME) for synergistic cancer immunotherapy. STING-stimulating cancer cells undergo immunogenic death (ICD) and are activated. When MONPs come into contact with acidic tumor tissue, they specifically dissociate and produce •OH in situ, which contributes to the ICD mechanism. Furthermore, Mn^2+^ triggered the STING and jointly stimulated the release of inflammatory cytokines and type I interferon to trigger certain T-cell responses. Concurrently, MONPs reduced Tregs and polarised M2 macrophages to the M1 type, which released a cascade adaptive immune response, relieving the immunosuppression of TME. MONPs have shown significantly greater effectiveness in halting tumor growth and averting lung metastases when combined with the anti-PD-1 antibody 117. This research indicates a promising approach for cancer immunotherapy by demonstrating the viability of using functional nanoparticles to enhance STING innate stimulation.

Nonetheless, potential improvements in the treatment of cancer can be achieved with Mn-based contrast agents for MRI-guided combinatory therapy. They are perfect for multimodal therapies because they can improve drug transport efficiency, catalyse therapeutic processes, and increase imaging precision, combining therapeutic and diagnostic capabilities for more effective, tailored cancer treatments.

## 4. Challenges of Mn-based CAs and their plausible remedies

Due to its unique magnetic properties and biocompatibility, iron (Fe) is a promising candidate for MRI contrast agents in cancer theranostics. It enhances imaging contrast, improving tumor visualization for early diagnosis and treatment monitoring. Fe-based agents can also be tailored to target specific cancer celIn MRI-guided photodynamic treatment (PDT), manganese-based (Mn-based) contrast agents are becoming more and more popular as dual-purpose materials. Because of the paramagnetic naturls, allowing for integrated diagnosis and therapy 118 . While iron (Fe) has notable advantages as an MRI contrast agent, there are also disadvantages compared to manganese (Mn)-based contrast agents in cancer theranostics 119. One major drawback of Fe is its limited relaxivity, which can result in less effective imaging at lower concentrations compared to Mn. Additionally, Fe-based agents may lead to the formation of aggregates or precipitation, which could affect their stability and distribution within the body. Conversely, Manganese has a higher atomic number, contributing to better signal intensity and contrast enhancement. Furthermore, Mn-based agents can offer more efficient cellular uptake and targeting capabilities, potentially improving diagnostic precision 120.

But still, some drawbacks are using Mn-based contrast agents such as the potential toxicity of excessive manganese accumulation, which can have a negative impact on the immune system and general health, which is one of the difficulties faced by Mn-based contrast agents (CAs). For extended in vivo use, it is also essential to guarantee their stability and biocompatibility because prolonged exposure to high manganese concentrations may result in undesirable side effects 121. Achieving the ideal balance between safety and therapeutic efficacy—especially when paired with other therapies like immunotherapy-remains a persistent obstacle for the clinical application of Mn-based CAs. Herein, we aim to explore such clinical challenges and thereby find a rationale to remediate them.

Wang and the team synthesized an Mn-chelate composed of O-carboxymethyl chitosan derivatives. This hybrid Mn-chelate-based MRI contrast agent shows significant enhancement in the image contrast in the case of the kidney and liver of Sprague Dawley (SD) rats. The in vitro and in vivo studies suggest that this compound is biocompatible and can be completely excreted from the SD rats within ten days of administration without any significant accumulation or toxicity 122.

Gene mutations that have significant pathogenic roles in Parkinsonism and the control of manganese transport and metabolism are present in these diseases. In manganese-related neurotoxicity, liver function is also crucial, and subclinical liver dysfunction may raise the risk of Parkinson's disease 123. Since mitochondria are an essential organelle for the synthesis of ATP, they are vulnerable to toxicity from Mn. Reduced energy production results from abnormal mitochondrial function caused by excess Mn. Gunter and associates demonstrated that ATP synthesis in mitochondria isolated from rat liver, heart, and brain could be inhibited by elevated Mn levels 124. Elevated intracellular Mn causes oxidative stress, mitochondrial failure, autophagy dysregulation, apoptosis, and protein dyshomeostasis, all of which impair regular cellular function and ultimately change neurotransmission. These lead to a range of neurological illnesses by disrupting regular neural function 125. Schrantz *et al.* found that Mn treatment caused dose- and time-dependent cell death in human B lymphocytes, activating the apoptotic marker proteins caspase-1 and 3 126. Neurotransmission may change dramatically as a result of Mn exposure. Zhang and colleagues observed a drop in dopaminergic (DAergic) neurons in the substantia par compacta (SNpc) and tyrosine hydroxylase (TH) protein in the rats that got Mn injection. This was linked to a decrease in dopamine (DA) and the D1 DA receptor 127.

Besides, folate-PEG-modified dihydroartemisinin (DHA) loaded manganese-doped mesoporous silica nanoparticles have been developed by Fei and his team to combat MRI-guided ferroptotic cancer therapy. The folate receptor herein acts as the binding molecule to the GSH-overexpressed cancer cells and thus produces enhanced MRI contrast 128. Hence, it can be said that scientists are working on microenvironment-based targeting moieties to prolong and enhance the contrast of the MRI images besides their clearance. It is observed that the designing approach may be attributed to the targeting efficiency of the contrast agents. In reality, Gallo *et al.* coated the MnO NPs with PEG and cRGDfK cyclopeptide and varied the chain length of the PEG to observe the targeting efficiency in tumors. It is found that the MnO NPs having a higher molecular weight (i.e., chain length) are more capable of targeting the tumor cells 129.

With the use of Mn^2+^ to reduce hypoxia and increase ROS production, Zhou's group created manganese-based theranostic nanoparticles for MRI-guided breast cancer treatment. Using glucose oxidase and manganese oxide, Korupalli *et al.* developed a multifunctional MnOx NSs@BSA-IR780-GOx nanocomposite that uses light-responsive dual-modal treatment to produce ROS in the tumor microenvironment 129. Such studies will surely unlock new avenues in the domain of MRI-imaging-guided theranostic modules.

Manganese-based MRI contrast agents in particular have problems with renal clearance and may be toxic to the liver and spleen due to Mn^2+^. Chevallier *et al.* addressed this by wrapping MnO NPs in a PEG-phosphonate dendron, which improved renal excretion and increased imaging potential 130. Currently, Mn-based MRI probes have been made of different biopolymers and other biomimetic systems having better renal clearance. However, Mn-based coordination complexes show enormous potential in this domain. We hope these coordination complexes and small molecules may be used in clinics in the near future.

The dual aptitudes of MnCAs in diagnostic and therapy make them highly promising for use in cancer theranostic applications. MnCAs are naturally eliminated by the body and show less toxicity than gadolinium-based drugs. By reducing *T1* relaxation time and enabling distinct tumor visualization, they improve MRI imaging 131. Targeting ligands, including peptides or antibodies, can be functionalized to MnCAs to enable selective accumulation in cancer cells. They are more effective in cancer theranostics when coated with biomimetic nanoparticles with cell membranes. Through homologous binding or immunological interactions, MnCAs can be cloaked in membranes from cancer or immune cells to improve tumor targeting, prolong circulation, and avoid immune detection 132. On Mn-based contrast agents, cell membrane coating promotes selective accumulation in tumor tissues, lowers toxicity, and increases biocompatibility. By producing ROS, this improves MRI imaging and therapeutic potential for the diagnosis and treatment of cancer.

By promoting osteoblast activity, collagen synthesis, and calcium absorption, manganese (Mn) aids in bone development. Mn can repair damaged bone tissue and deliver therapeutic drugs by being included in scaffolds or nanoparticles used in bone cancer treatment. Materials containing Mn also improve imaging to detect cancer and track its progress throughout therapy. For instance, a multifunctional nanoplatform based on PAH-MnO_2_@BP nanosheets loaded on thermosensitive gel poly(d,l-lactide)-poly(ethylene glycol)-poly(d,l-lactide) (PDLLA-PEG-PDLLA, PLEL) for *in situ* osteosarcoma synergy therapy (photothermal/photodynamic/chemotherapy, PTT/PDT/CHT), was developed by Fu *et al.* 133. By turning excess hydrogen peroxide into oxygen, manganese dioxide on BP NSs improves photodynamic treatment (PDT) and decreases tumor hypoxia. The photothermal effect of BP NSs also controls the release of doxorubicin. The hydrogel composite optimizes tumor efficacy while reducing relapse by integrating photothermal therapy (PTT), photodynamic therapy (PDT), and chemotherapy (CHT). This strategy has the potential to treat osteosarcoma and provide individualized cancer care.

Moreover, since the late 1980s, neurotoxicity has been a great concern for mankind. It is the damage caused by toxic substances or conditions to the nervous system, potentially leading to temporary or permanent impairment of neural function. Neurotoxicity can arise from various sources such as exposure to heavy metals (e.g., manganese, lead, mercury), pesticides, solvents, industrial chemicals overdose, or chronic use of certain drugs, etc. Paramagnetic properties of the Manganese make manganese-enhanced MRI (MEMRI) a useful tool for neuroimaging since it makes it possible to observe the activity and structure of neurons. However, because of its ability to build up in the brain and disrupt regular neuronal function, manganese (Mn) neurotoxicity is still a serious problem. The severity of Mn toxicity depends on the dose, mode of administration, and species-specific sensitivity. It can cause severe neurological effects, such as motor and mental disorders. MEMRI offers valuable insights into neural function, but minimizing neurotoxic risks requires careful consideration of dosing and species-specific differences. Researchers and clinicians should balance its benefits against potential adverse effects by following safety guidelines and using the lowest effective doses 134.

Nonetheless, it takes several steps to translate MnCAs for cancer theranostic applications into clinical use. To begin with, comprehensive preclinical research has to validate safety, biocompatibility, and therapeutic and imaging efficacy. To reduce toxicity, MnCA formulations must be optimized to target certain tumors and ensure effective renal clearance 135. Evaluation of patient outcomes, treatment efficacy, diagnostic accuracy, and cooperation between researchers, physicians, and industry should be the main goals of clinical studies for translating MnCAs from the lab to the clinic.

## 5. Summary

Manganese (Mn)-based MRI contrast agents have made significant contributions to cancer therapy by offering enhanced imaging capabilities, superior biocompatibility, and targeted delivery to cancerous tissues. Their strong paramagnetic properties improve *T1*-weighted imaging, enabling better differentiation between healthy and diseased tissues, while their natural role as an essential trace element reduces the risks of toxicity. Mn-based agents also demonstrate a unique ability to target specific cellular pathways, such as calcium transport, facilitating precise tumor localization and monitoring of therapeutic responses. However, some major concerns regarding manganese ions need to be addressed to make them efficient MRI contrast agents. First, the neurotoxicity of Mn^2+^ should be controlled by encapsulating them into suitable hosts. Manganese complexes and chelates can be formed with organic and inorganic complexes for this purpose. Second, the use of biomimetic and biocompatible probes must be explored. Numerous research groups are currently working in this field, making manganese-based probes a more feasible alternative. Third, pharmacokinetic/pharmacodynamic profiles should be investigated before deploying such theranostic agents. The accumulation of manganese in the brain and other organs could worsen patients' health conditions. Therefore, proper excretion profiles of these agents must be determined well in advance. Last, but not least, more work is needed to achieve better *T1* relaxation in Mn-based contrast agents. So far, Mn-based agents cannot qualify as contrast agents like Gd due to the lower number of spin quantum states available in the Mn ions. However, the future research could also focus on optimizing Mn-chelated complexes for greater stability and specificity, developing multimodal Mn-based agents for combined diagnostic and therapeutic (theranostic) applications, and exploring nanoformulations to improve biodistribution and controlled release. This will not only position Mn-based contrast agents as an alternative to commercial Gd-based agents but also potentially make them superior in many aspects.

## Figures and Tables

**Table 1:** The comparison of the *r1* relaxivity of typical gadolinium agents compared to manganese-based contrast agents where it shows that the Mn-based agents achieved a higher *r1* relaxivity rate than Gd agents

Sample name	*r_1_* relaxivity (mM^-1^ s^-1^)	References
MnO-PEG-RGD	12.1	17
Mn_0.5_Mg_2.6_Al_1_-LDH	7.60	18
Mn_3_Fe_1_-LDH	7.83	19
MnO*_x_*-SiO_2_ hollow@PEG	8.81	20
MnO@Fe_3_O_4_-OH-PEG-PH	22.8	21
Gd_2_O_3_	9.14	22
GdDTPA- conjugated Fe_3_O_4_@SiO_2_@mSiO_2_	6.13	23
MnO-PEG-AS1411 Aptamer	12.9	24
Gd-DOTA	4.2	25
Mn-M48SN	8.4	26
Polyacrylic acid-coated MnO nanoparticles	9.3	27
USMnO@ZDS	15.6	28
Gd-DOTA	5.77	29
